# Protective effects of 3-(4-hydroxy-3-methoxyphenyl) propionic acid against dexamethasone-induced muscle atrophy: modulation of associated genes and oxidative stress in female mice

**DOI:** 10.1016/j.bbrep.2026.102483

**Published:** 2026-02-09

**Authors:** Anayt Ulla, Honomi Ogura, Md Mizanur Rahman, Saya Nakamura, Yuka Ichiba, Haruka Tsuda, Takayuki Uchida, Hiroyuki Kayaki, Yosuke Nishitani, Susumu Yoshino, Hiroshige Kuwahara, Toshiro Matsui, Takeshi Nikawa

**Affiliations:** a Department of Nutritional Physiology, Institute of Biomedical Sciences, Tokushima University Graduate School, 3-18-15 Kuramoto-cho, Tokushima, 770-8503, Japan; b Department of Bioscience and Biotechnology, Faculty of Agriculture, Graduate School of Kyushu University, 744 Motooka, Fukuoka, 819-0395, Japan; c Research Center, Maruzen Pharmaceuticals Co., Ltd., 1089-8 Sagata Shinichi-Cho Fukuyama-City, Hiroshima, 729-3102, Japan

**Keywords:** Glucocorticoid, Oxidative stress, Ubiquitin ligase, Muscle atrophy, 3-(4-hydroxy-3-methoxyphenyl) propionic acid, 4-Hydroxy-3-methoxycinnamic acid

## Abstract

Muscle atrophy is a growing concern, particularly in older adults and people with sedentary lifestyles. Because treatment options are limited, extensive research is crucial to discover novel therapeutic agents. Thus, we investigated the effect of 3-(4-hydroxy-3-methoxyphenyl) propionic acid (HMPA) and its parent compound, 4-hydroxy-3-methoxycinnamic acid (HMCA), on dexamethasone (Dex)-induced muscle atrophy in C57BL/6J female mice. Dex injection (10 mg/kg body weight [BW] in mice for 10 consecutive days negatively affected body weight, gastrocnemius and tibialis anterior muscle mass, myofiber cross sectional area (CSA) and level of myosin heavy chain (MyHC) protein. Atrogin-1 and muscle ring finger protein-1, two major muscle atrophy-associated ubiquitin ligases, were significantly increased following Dex administration, along with their upstream regulators forkhead box O3a (FoxO3a) and Krüppel-like factor 15 (KLF15). Furthermore, Dex-induced oxidative stress by increasing malondialdehyde and advanced oxidation protein products in plasma and skeletal muscle. Intriguingly, HMPA and HMCA administration (50 mg/kg BW) for 21 days effectively prevented the attenuation of muscle mass, myofiber CSA and MyHC protein levels and suppressed ubiquitin ligase expression by ameliorating the upstream transcriptional factors FoxO3a and KLF15. Moreover, increased oxidative stress and oxidative stress-sensitive casitas B-lineage lymphoma proto-oncogene-b (Cbl-b) ubiquitin ligase induced by Dex were effectively diminished by HMPA/HMCA administration. These observations suggest that HMPA and HMCA may be potential *in vivo* therapeutic agents that attenuate muscle atrophy by reversing atrophy-mimicking genes, oxidative stress, and related anomalies.

## Highlights

• HMPA and HMCA effectively attenuated Dex-mediated muscle atrophy.
• Both HMPA and HMCA are effective in suppressing Atrogin-1, MuRF-1 and Cbl-b.
• Oxidative stress-mediated pathways of muscle atrophy also suppressed by both compounds.
• HMPA and HMCA could be potential therapeutic agents to reduce Dex-induced side effects.

## 1 Introduction

Skeletal muscle is the largest secretory and metabolic organ in the body. It plays an indispensable role in interorgan communication with tissues, such as bone, nerves, and the liver, and acts as the principle site for glycogenesis and a protein reservoir to regulate energy and protein balance [1]. Diabetes, cardiovascular disease, and higher mortality rates are closely associated with skeletal muscle dysfunction [2]. Skeletal muscle atrophy refers to muscular dysfunction involving an imbalance between muscle protein synthesis and breakdown, resulting in the loss of muscle mass and strength. This condition is triggered by various factors, including immobilization, disuse, aging, denervation, cachexia, and glucocorticoids [3]. Glucocorticoids are usually prescribed to alleviate inflammatory diseases, like rheumatoid arthritis, asthma, and multiple sclerosis, and are also immunosuppressants. However, indiscriminate use leads to detrimental side effects, including osteoporosis, adrenal gland dysfunction, hyperglycemia, and muscle atrophy [4]. Because treatments for skeletal muscle atrophy are currently limited, novel therapeutic targets and agents to modulate skeletal muscle atrophy are an urgent priority.

Dexamethasone (Dex), a potent synthetic glucocorticoid, disrupts the equilibrium between muscle protein synthesis and degradation [5]. The ubiquitin-proteasome system (UPS) is a ubiquitin-mediated protein degradation mechanism and serves as the major pathway for Dex-induced proteolysis. Upon binding to its receptor, Dex instigates the expression of the transcription factor Krüppel-like factor 15 (KLF15) [6], which triggers the transcription of genes associated with muscle atrophy, enhancing the expression of proteins MAFbx1/Atrogin-1 and muscle ring finger protein-1 (MuRF-1) [7]. Dex has also been reported to prompt oxidative stress in both *in vitro* and *in vivo* muscle atrophy models [[8], [9], [10]]. Casitas B-lineage lymphoma proto-oncogene-b (Cbl-b) is an oxidative stress-sensitive ubiquitin ligase [11] that leads to the degradation of insulin receptor substrate-1 (IRS-1) through the ubiquitination process [12]. IRS-1 malfunction further contributes to the interruption of the insulin-like growth factor-1 (IGF-1) signaling pathway, resulting in increased expression of Atrogin-1 and MuRF-1 through forkhead box O3a (FoxO3a) dephosphorylation [13,14]. Therefore, canonical and oxidative stress-mediated pathways represent potential therapeutic targets for exploring Dex-induced muscle atrophy.

The widely distributed polyphenol 3-(4-hydroxy-3-methoxyphenyl) propionic acid (HMPA) is an *in vivo* metabolite of 4-hydroxy-3-methoxycinnamic acid (HMCA) and has attracted attention in the fields of biochemistry and pharmacology due to its diverse biological activities. It is found in cocoa and cocoa products, coffee, tea, fruits, legumes, and nuts [15]. The gut microbiota metabolizes many phenolic acids and flavonoids into HMPA [16]. HMPA possesses various pharmacological characteristics, including antioxidant, anti-inflammatory, anti-obesity, neuroprotective, hepatoprotective, antidiabetic, and anticancer properties, and it also enhances cognitive function and muscle strength [[17], [18], [19]]. HMCA is the parent compound of HMPA. It is extensively metabolized into HMPA and has been detected in the biological systems of rodents following dietary intake [16]. Moreover, the beneficial effects of HMCA have been attributed to HMPA in previous studies where treatment with drugs interfering with gut metabolism abolished the beneficial effects of HMCA [16]. We previously reported the beneficial effects of HMPA in Dex-induced muscle atrophy in C2C12 myotubes [15], but the *in vivo* effect of HMPA on glucocorticoid-induced muscle atrophy remains unknown. Thus, this study investigated the impact of HMPA and HMCA on Dex-induced skeletal muscle atrophy in an *in vivo* model using C57BL/6J mice treated with a high (50 mg/kg BW) or low (5 mg/kg BW) dose of HMPA or a high (50 mg/kg BW) dose of HMCA to elucidate the effect of both compounds.

## 2 Materials and methods

### 2.1 Reagents and chemicals

HMPA and HMCA were generously provided by Maruzen Pharmaceuticals Co., Ltd. (Hiroshima, Japan). Dex was purchased from Sigma-Aldrich (St. Louis, MO, USA), thiobarbituric acid and tetramethoxypropane from Tokyo Chemical Industry (Tokyo, Japan), and potassium iodide and chloramine-T from Nacalai Tesque (Kyoto, Japan). 1-(or *N*_τ_)*-*methyl-L-His (Lot: BCCD8667) and 3-(or *N*_π_)-methyl-L-His (Lot: BCCG7.84) were purchased from Sigma-Aldrich, and 3-(or *N*_π_-) methyl-l-histidine-*d*3 (Lot: 249,811) was sourced from MedChemExpress (Monmouth Junction, NJ, USA). Distilled water, acetonitrile, formic acid, and ammonium formate (all LC–MS grades) were purchased from Merck KGaA (Darmstadt, Germany). All other reagents were of analytical grade and used without further purification.

### 2.2 Animals and experimental designs

We obtained 30 female C57BL/6J mice (age 12–13 weeks, weight 21–22 g) from Japan SLC (Shizuoka, Japan). We used female mice because of their robust responsiveness to Dex and the low effect of anabolic testosterone [20,21]. They were housed individually in cages under a constant temperature of 20 –25 °C with a 12-h dark/light cycle and given free access to standard laboratory food and water throughout the experiment. Following acclimatization for 1 week, the mice were randomly divided into five experimental groups (n = 6 per group) as follows: group I, control; group II, dexamethasone (Dex); group III, Dex + HMPA5; group IV, Dex + HMPA50; and group V, Dex + HMCA50. HMPA and HMCA were dissolved in normal saline, and fresh solutions were prepared daily for administration via oral gavage as specified for each group. The mice in group I were administered normal saline (vehicle for HMPA and HMCA) via oral gavage (in a volume equal to the HMPA/HMCA solution administered to other groups) and injected intraperitoneally with MilliQ water (vehicle for Dex). The mice in group II were administered normal saline via oral gavage, group III were administered HMPA (5 mg/kg body weight [BW]), group IV were administered HMPA (50 mg/kg BW), and group V were administered HMCA (50 mg/kg BW) during the 21-day experimental period. A single 50 mg/kg dose of HMCA was chosen to achieve sufficient systemic exposure, given its rapid microbial conversion to HMPA [16], and to match the upper HMPA dose for direct comparison. The mice in groups II, III, IV, and V were injected with Dex (10 mg/kg BW) daily for the last 10 days of the experimental period to induce muscle atrophy. All mice were weighed daily throughout the 21-day experiment.

### 2.3 Ethical approval

Ethical approval for this study was granted by the Tokushima University Committee on Animal Experiments with the approval number T2021-107 (03/30/2022-03/30/2025). All procedures were performed according to the university's guidelines for the care and handling of laboratory animals.

### 2.4 Animal sacrifice and sample collection

After the final Dex and/or HMPA and HMCA feeding, the mice fasted for 6 h and were sacrificed for blood and tissue harvesting. Blood was collected in EDTA-coated tubes by cardiac puncture. Following centrifugation at 8000 rpm (5870×*g*) for 15 min at 4 °C, the plasma was separated and stored at −80 °C for further analysis. The gastrocnemius (GA), tibialis anterior (TA), extensor digitorum longus (EDL), and soleus muscles were harvested, weighed, and stored at −80 °C for further analysis.

### 2.5 Cross-sectional area (CSA) analysis

After euthanasia, the gastrocnemius (GA) muscles were rapidly excised, gently positioned on wooden blocks using tissue adhesive, and immediately frozen in isopentane pre-cooled with liquid nitrogen. The frozen muscles were sectioned at a thickness of 10 μm using a cryostat (LEICA- CM1850, Leica Biosystem, Germany). The sections were stained with hematoxylin for 1 min followed by eosin for 3 min at room temperature, then rinsed and mounted for imaging.

Muscle histology was captured using a phase-contrast microscope (BIOREVO BZ-9000; Keyence, Osaka, Japan), and images were analyzed with BZ-II Analyzer software (Keyence). For cross-sectional area (CSA) quantification, ten randomly selected fields per muscle were imaged at 20 × magnification in a blinded manner. Each field contained approximately 100–300 fibers (fiber area range = 100–5000 μm^2^), yielding a total of about 1000–3000 fibers analyzed per muscle. Images were collected from multiple regions of each GA muscle to ensure representative sampling across the tissue.

### 2.6 Western blot

The GA muscle tissue samples were homogenized in a lysis buffer (10:1 buffer-to -tissue ratio) composed of 50 mM Tris HCl (pH 7.5), 150 mM NaCl, 5 mM EDTA, 10 mM NaF, 2 mM Na_3_VO_4_, 1% Triton X-100, protease inhibitor cocktail (Roche Diagnostics, Rotkreuz, Switzerland), and 10 μM MG-132. The homogenate was centrifuged at 12,000×*g* for 15 min at 4 °C, and the supernatant was collected for protein quantification using a Pierce™ BCA protein assay kit (Thermo Fisher Scientific, Waltham, MA). For Western blot analysis, the ProteinSimple™ WES system (Protein Simple, CA, USA) was employed following the manufacturer's guidelines. Briefly, 1 μg protein was combined with Simple Western sample buffer and 5 × Fluorescent Master Mix, then denatured at 95^ο^C for 5 min. Protein samples, primary and secondary antibodies, and chemiluminescent substrate were loaded into their respective wells in the Simple WES microplate. The plate was then inserted into the WES instrument, along with the capillary cartridge to carry out automated Western blotting. The process was completed in approximately 3 h, after which the software-generated data were analyzed to quantify protein expression levels. Manual western blotting was also performed to detect phosphorylated Akt levels following our previously published method [8]. Antibodies for fast-type myosin heavy chain (MyHC) (1:100) (M4276) (Sigma-Aldrich, Missouri, USA); β-actin (1:200) (AC004) (ABclonal, MA, USA); slow-type MyHC (1:100) (NBP2-50298) (Novus Biologicals, Littleton, CO, USA); MAFbx/Atrogin-1 (1:200) (ab168372), MuRF-1 (1:200) (ab77577) (Abcam, Cambridge, UK); Cbl-b (1:200) (9498S), IRS-1 (1:100) (3407S), total FoxO3a (1:100) (2497S), phosphorylated FoxO3a (1:100) (9466S), total Akt (1:100) (9272) and phosphorylated-Akt^Thr308^ (1:1000) (9275S), rabbit IgG (1:200/2000) (7074P2), anti-mouse IgG (7076S) (Cell Signaling Technology, Danvers, MA, USA) were used.

### 2.7 Reverse transcription-quantitative polymerase chain reaction (qPCR)

The isolation of total RNA from GA muscle homogenates was performed with ISOGEN™ (Nippon Gene, Tokyo, Japan), followed by RNA concentration measurement using a Nanodrop 1000 spectrophotometer (Thermo Fisher Scientific). Next, the extracted 1 μg RNA underwent reverse transcription into cDNA (PrimeScript IV cDNA synthesis Mix, Takara, Japan). Then, qPCR was performed using SYBR Green dye (*Power*SYBR Green PCR Master Mix, Thermo Fisher Scientific), and appropriate primers on a StepOnePlus Real-Time PCR System (Applied Biosystems, CA, USA). 18S ribosomal RNA was used as the internal standard. Table 1 lists the PCR primer sequences.

**Table 1:** Primers used for PCR.

Target gene		Sequence	Length (bp)
MAFbx1/Atrogin-1	S	GGCGGACGGCTGGAA	101
	AS	CAGATTCTCCTTACTGTATACCTCCTTGT	
MuRF-1	S	TGTCTGGAGGTCGTTTCCG	183
	AS	CTCGTCTTCGTGTTCCTTGC	
Cbl-b	S	GAGCCTCGCAGGACTATGAC	222
	AS	CTGGCCACTTCCACGTTATT	
Catalase	S	ATGGCTTTTGACCCAAGCAA	69
	AS	CGGCCCTGAAGCTTTTTGT	
KLF-15	S	CCAGGCTGCAGCAAGATGTACAC	125
	AS	TGCCTTGACAACTCATCTGAGCGG	
Nrf2	S	AGGACATGGAGCAAGTTTGG	482
	AS	TCTGTCAGTGTGGCTTCTGG	
18Sr	S	CATTCGAACGTCTGCCCTA	119
	AS	CCTGCTGCCTTCCTTGGA	

18Sr, 18S ribosomal RNA; Cbl-b, Casitas B-lineage lymphoma proto-oncogene-b; GR, glucocorticoid receptor; KLF15, Krüppel-like factor 15; MuRF-1, muscle ring finger protein-1; Nrf2, Nuclear factor erythroid 2-related factor 2.

### 2.8 Measurement of methyl-His levels

1-methyl-L-His and 3*-*methyl-L-His are two methylhistidine isomers that have been studied in the context of muscle metabolism, particularly concerning muscle atrophy. We determined the plasma levels of 1*-*methyl-L-His and 3-methyl-L-His by mixing an aliquot (10 μL) of the plasma sample with 80 μL distilled water, followed by the addition of 10 μL of 10 μM 3-methyl-L-His-*d*_3_ as an internal standard (IS; final concentration: 1 μM). The solution was centrifuged at 12,000×*g* for 10 min at 4 °C, and the supernatant underwent ultrafiltration using an Amicon Ultra Centrifugal Filter (0.5 mL, MWCO 3 kDa; Merck Millipore Ltd., Tullagreen, Ireland) at 14,000×*g* for 30 min at 4 °C. The resultant filtrate was subjected to ultra-performance liquid chromatography coupled with triple quadrupole mass spectrometry (UPLC-QqQ/MS).

Chromatographic separation was performed using an ACQUITY UPLC System (Waters Co., Milford, MA, USA), and LC separation was performed on a Scherzo SS-C18 column (2.0 I.D. × 50 mm, 3 μm; Imtakt Co., Kyoto, Japan) with a gradient elution of solution A (0.1% formic acid) to solution B (50 mM ammonium formate in 50% acetonitrile) at a flow rate 0.5 mL/min at 40 °C as follows: 0–3 min, 1% B; 3–13 min, 1%–30% B; 13–16 min, 30%–100% B; 16–19 min, 99% B; 19–21 min, 99%–1% B; and 21-30 min, 99% B.

The MS analysis was performed using a Xevo TQD Triple Quadrapole Mass Spectrometer (Waters Co.) in the electrospray ionization (ESI)-positive ion mode ([M + H]^+^ for 1*-* and 3-methyl-L-His, 170.1 *m/z*; [M + H]^+^ for 3-methyl-L-His-*d*_3_, 173.1 *m/z*). The ESI-QqQ/MS conditions were as follows: capillary voltage, 2.5 kV; cone voltage, 22 V; and desolvation (nitrogen) gas, 1200 L/h at 650 °C and 50 L/h for the cone. The MS data were analyzed using MassLynx v4.1 software (Waters Co.). The plasma concentration of 1- or 3-methyl-L-His was calculated using a calibration curve for each analyte by IS-aided UPLC-QqQ/MS: 1-methyl-L-His: y = 0.952*x* + 0.101 (0–30 μmol/L, R^2^ = 0.9993) and 3-methyl-L-His: y = 0.706*x*−1.12 (0–70 μmol/L, R^2^ = 0.9903), where *x* is the analyte concentration and *y* is the ratio of the observed peak area of each analyte against the IS (3-methyl-L-His-*d*_3_).

### 2.9 Analysis of oxidative stress markers

The GA muscle was homogenized using lysis buffer in the same manner as for Western blot preparation (described above) and centrifuged at 12,000×*g* for 15 min at 4^ο^ C. Then, the clear liquid phase was collected for calorimetric measurement of malondialdehyde (MDA) and advanced oxidation protein products (AOPP). We measured the MDA and AOPP levels in plasma and GA muscle lysates using a thiobarbituric acid reactive substance colorimetric assay and a colorimetric method, respectively, as described by Ulla et al. [22]. The AOPP concentration was reported in nmol/mL^−1^ or per mg chloramine-T equivalents based on the chloramine-T standard curve. The curve demonstrated a linear regression between concentration and absorbance (340 nm) within the range of 0 –100 nmol/mL.

### 2.10 Statistical analysis

Data were presented as means ± standard error of the mean (SEM). Comparisons among groups were performed using One-way analysis of variance (ANOVA) followed by Tukey's post hoc multiple comparison test. Statistical analyses were conducted with GraphPad Prism version 9.3.1 (GraphPad Software, San Diego, CA, USA). P-values <0.05 were considered statistically significant.

## 3 Results

### 3.1 Effect of HMPA and HMCA treatment on body weight

**Fig. 1:**
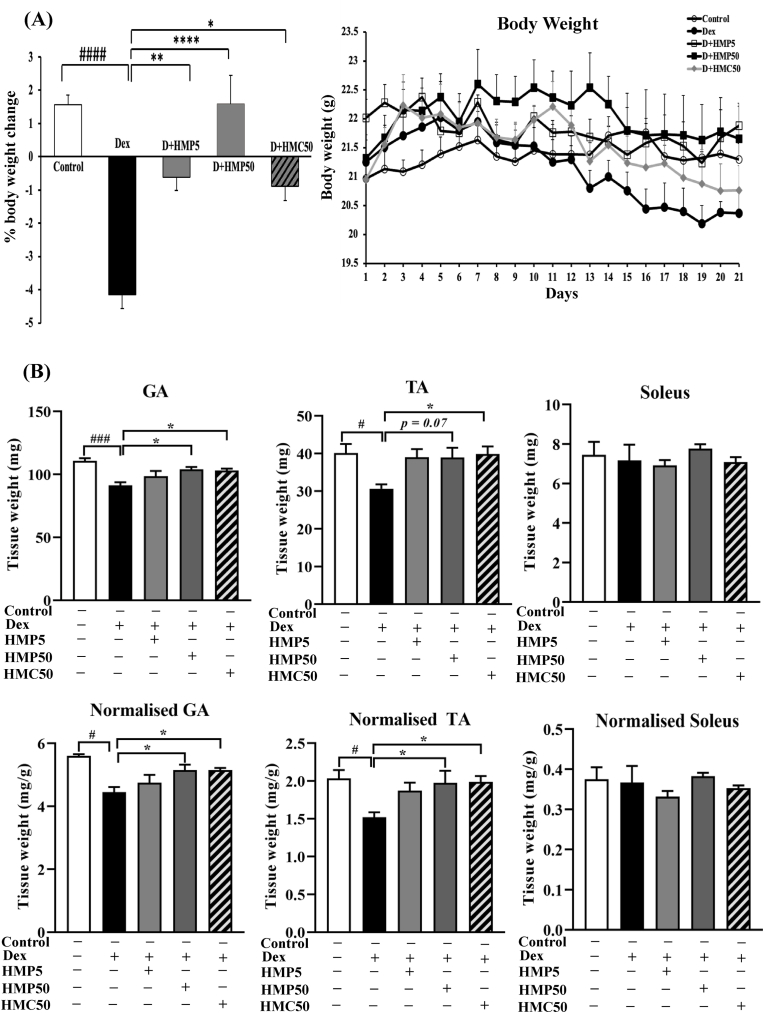
**HMPA and HMCA ameliorated the Dex-induced loss of body and muscle weight in mice.** (A) Changes in body weight of experimental mice. Mice were treated with Dex (10 mg/kg BW) and/or HMPA (5 or 50 mg/kg BW) or HMCA (50 mg/kg BW) for the indicated durations, and body weights were measured daily. (B) Total weight (upper panel) and normalized weight (lower panel) of the GA, TA, and soleus muscles. Normalized weight = total muscle weight/body weight. Data are expressed as mean ± SEM (n = 6/group). Statistical analysis was performed using one-way ANOVA followed by Tukey's post hoc test. ∗P < 0.05, ∗∗P < 0.01, ∗∗∗P < 0.001 vs. Dex group; #P < 0.05, ##P < 0.01, ###P < 0.001 vs. Control group. Dex, dexamethasone; HMP5/HMP50/HMC50 indicates HMPA (5 mg/kg BW)/HMPA (50 mg/kg BW)/HMCA (50 mg/kg BW); D + HMP5/HMP50/HMC50 indicates Dex + HMPA (5 mg/kg BW)/HMPA (50 mg/kg BW)/HMCA (50 mg/kg BW); GA, gastrocnemius; TA, tibialis anterior; BW, body weight.

The Dex-treated mice weighed considerably less than the control mice (Fig. 1A). HMPA treatment with the higher dose (50 mg/kg BW) prevented body weight loss, and the mice in this group resembled those in the control group. However, the lower dose of HMPA (5 mg/kg BW) and HMCA only partially protected against body weight loss compared with the Dex-treated mice (Fig. 1A).

### 3.2 Effect of HMPA and HMCA treatment on muscle weight

At the end of the study, the total and normalized weight (muscle weight/BW) of the GA and TA muscles were significantly reduced in the Dex-treated mice compared with the vehicle-treated control mice (Fig. 1B), but the weight of the soleus and EDL (data not shown) muscles was unaffected by the Dex treatment. Treatment with the higher dose of HMPA and HMCA significantly attenuated GA and TA muscle weight loss induced by Dex. Both the total and normalized weights of the GA and TA muscles were restored in mice cotreated with Dex and HMPA/HMCA compared with Dex-only treated mice (Fig. 1B). These findings suggested that the higher dose of HMPA and HMCA might be an effective phytochemical attenuator of muscle atrophy.

### 3.3 The effect of HMPA and HMCA on the CSA of myofibers

**Fig. 2:**
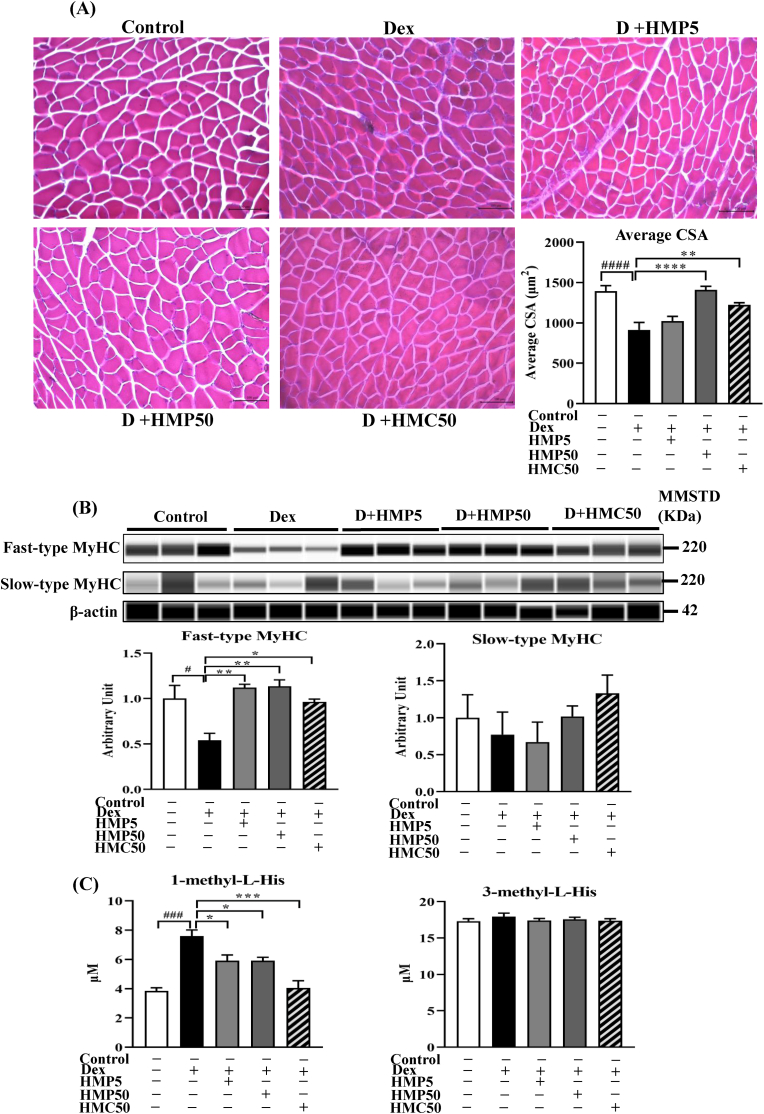
**HMPA and HMCA attenuated Dex-induced myofiber CSA and muscle protein degradation in mice.** Mice were treated with Dex (10 mg/kg BW) and/or HMPA (5 or 50 mg/kg BW) or HMCA (50 mg/kg BW) for the indicated durations. (A) H&E staining of GA muscle. (B) Expression levels of fast- and slow-type myosin heavy chain (MyHC) protein in GA muscle samples. Representative lane views obtained from the automated capillary ProteinSimple WES system are shown. Data are expressed as mean ± SEM (n = 3/group). (C) Levels of 1-methyl-L-His and 3-methyl-L-His in GA muscle samples. Data are expressed as mean ± SEM (n = 5/group). Statistical analysis was performed using one-way ANOVA with Tukey's post hoc test. ∗P < 0.05, ∗∗P < 0.01, ∗∗∗P < 0.001 vs. Dex group; #P < 0.05, ##P < 0.01, ###P < 0.001 vs. Control group. Dex, dexamethasone; HMP5/HMP50/HMC50 shows HMPA (5 mg/kg BW)/HMPA (50 mg/kg BW)/HMCA (50 mg/kg BW); MMSTD, molecular mass standard.

To assess the effects of HMPA and HMCA on muscle fiber size in dexamethasone-treated mice, hematoxylin and eosin (H&E) staining was performed on cross-sections of gastrocnemius (GA) muscle tissue (Fig. 2A). Dexamethasone administration markedly reduced myofiber cross-sectional area (CSA) compared to the control group, indicating significant muscle atrophy. Notably, co-treatment with high doses of HMPA (50 mg/kg) or HMCA (50 mg/kg) substantially mitigated this reduction in CSA, suggesting protective effects against dexamethasone-induced muscle wasting. Quantitative analysis confirmed that the average CSA was significantly preserved in these treatment groups compared to Dex alone.

### 3.4 Effect of HMPA and HMCA treatment on muscle protein degradation

Dex increases MuRF-1 expression and preferentially affects fast-type MyHC while exerting less impact on slow-type MyHC [23]. We investigated the expression of fast- and slow-type MyHC in GA muscle following Dex and HMPA/HMCA treatment. The mice treated with Dex showed significantly reduced levels of fast-type MyHC protein compared with the vehicle-treated control mice (Fig. 2B). Both HMPA and HMCA treatment effectively attenuated the Dex-mediated degradation of fast-type MyHC. In contrast, there were no comparable changes in the levels of slow-type MyHC protein among the different experimental groups (Fig. 2B). These observations suggested that Dex significantly affected fast- but not slow-type MyHC protein and that both HMPA and HMCA were effective attenuators of Dex-induced proteolysis of MyHC.

We measured methyl-L-His, a biomarker of muscle protein metabolism and degradation. Although no significant changes were noted in the concentration of 3-(or *N*_π_)-methyl-L-His, 1-(or *N*_τ_)*-*methyl-L-His was significantly increased following Dex treatment compared with the control mice (Fig. 2C). Interestingly, HMPA and HMCA effectively suppressed the increased 1-(or *N*_τ_)*-*methyl-L-His induced by Dex. This observation further supported the effectiveness of HMPA and HMCA against Dex-induced proteolysis of MyHC.

### 3.5 Effect of HMPA and HMCA on muscle atrophy-associated E3 ubiquitin ligases

**Fig. 3:**
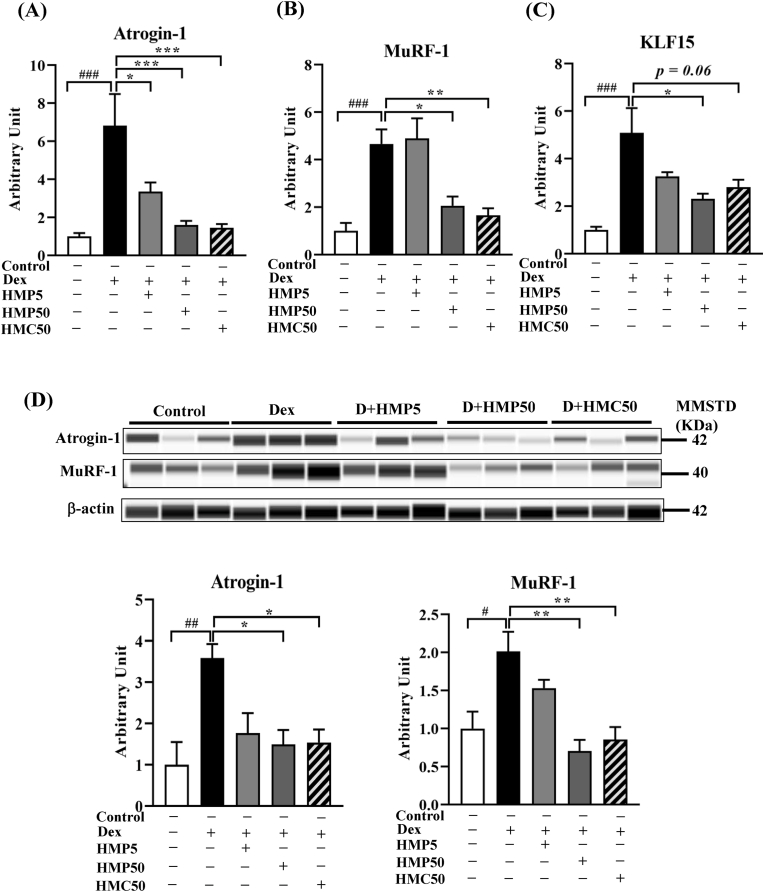
**HMPA and HMCA suppressed Dex-induced expression of ubiquitin ligases and upstream mediator KLF15.** Mice were treated with Dex (10 mg/kg BW) and/or HMPA (5 or 50 mg/kg BW) or HMCA (50 mg/kg BW) for the indicated durations. (A–C) mRNA expression levels of Atrogin-1, MuRF-1, and KLF15 (n = 5/group). (D) Protein levels of Atrogin-1 and MuRF-1 (n = 3/group). Representative lane views obtained from the automated capillary ProteinSimple WES system are shown. Data are expressed as mean ± SEM. Statistical analysis was performed using one-way ANOVA followed by Tukey's post hoc test. ∗P < 0.05, ∗∗P < 0.01, ∗∗∗P < 0.001 vs. Dex group; #P < 0.05, ##P < 0.01, ###P < 0.001 vs. Control group. Dex, dexamethasone; HMP5/HMP50/HMC50 indicates HMPA (5 mg/kg BW)/HMPA (50 mg/kg BW)/HMCA (50 mg/kg BW); KLF15, Krüppel-like factor 15; MuRF-1, muscle ring finger protein-1; MMSTD, molecular mass standard.

We investigated mRNA and protein expression of the muscle atrophy-associated ubiquitin ligases Atrogin-1 and MuRF-1 in the GA muscle. Both the mRNA and protein levels of Atrogin-1 and MuRF-1 were significantly increased following Dex treatment compared with the vehicle-treated control mice (Fig. 3A, B, and D). Interestingly, treatment with the higher dose of HMPA and HMCA effectively suppressed the Dex-induced upregulation of ubiquitin ligases. Treatment with the lower dose of HMPA tended to decrease the ubiquitin ligase level compared with the Dex group.

Next, we investigated the mRNA expression of KLF15, the upstream regulator of Atrogin-1 and MuRF-1 and a direct target of the glucocorticoid receptor. Compared with the vehicle-treated control mice, Dex treatment significantly increased KLF15 expression (Fig. 3C). Treatment with the higher dose of HMPA and HMCA reversed the Dex-mediated increase in the KLF15 level, along with minor suppressive effects caused by the lower dose of HMPA (5 mg/kg BW) (Fig. 3C). These results indicated that HMPA and HMCA regulated Atrogin-1 and MuRF-1 expression by modulating KLF15 expression.

### 3.6 Effect of HMPA and HMCA on the oxidative stress-sensitive Cbl-b ubiquitin ligase

**Fig. 4:**
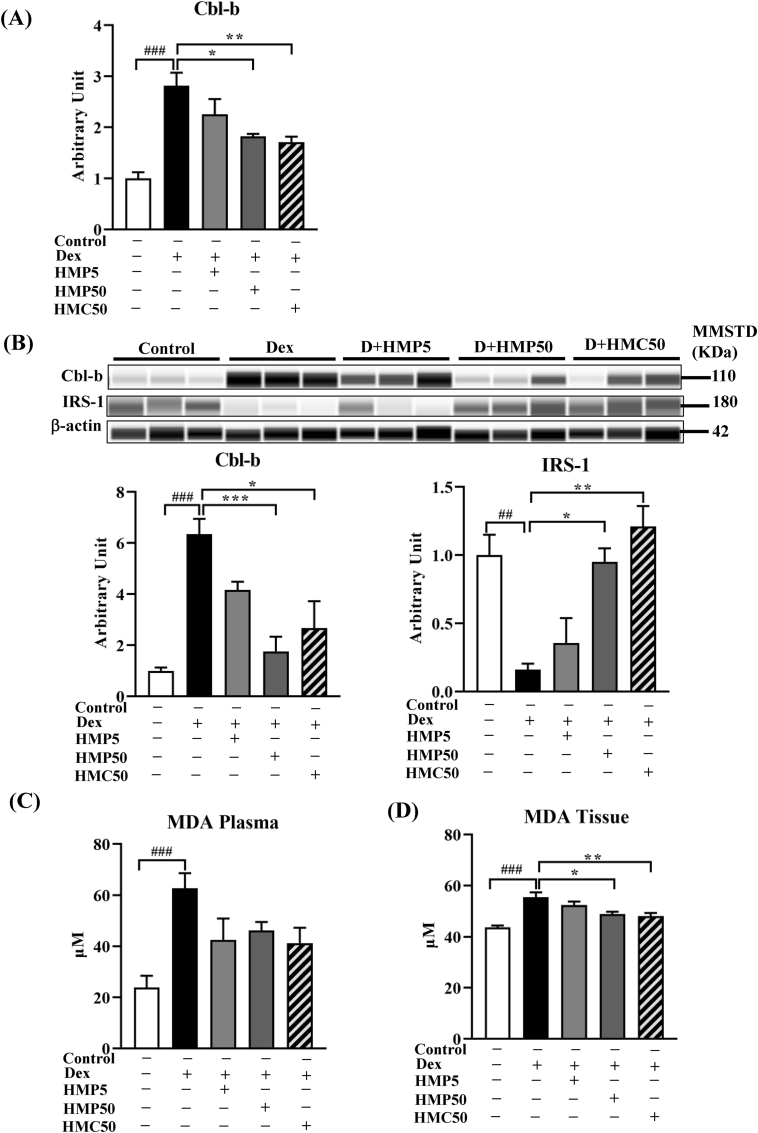

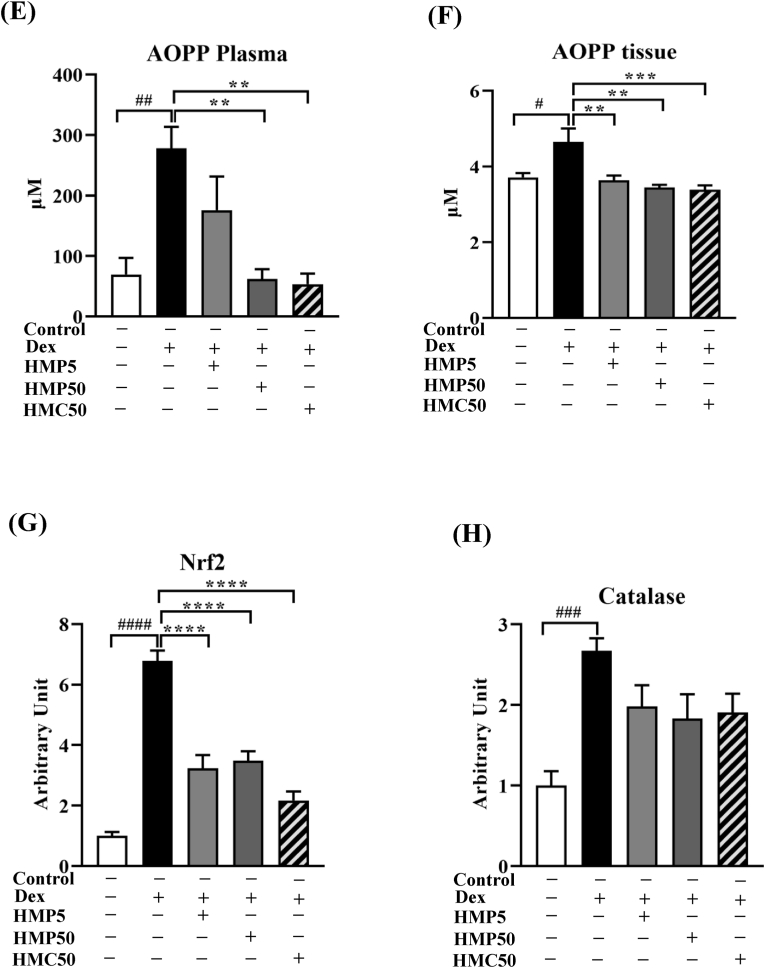
**HMPA and HMCA attenuated Dex-induced Cbl-b expression.** Mice were treated as described above and assessed for mRNA and protein expression of the oxidative stress–sensitive ubiquitin ligase Cbl-b. (A) mRNA expression of Cbl-b (n = 5/group). (B) Protein levels of Cbl-b and IRS-1 in GA muscle (n = 3/group). Representative lane views obtained from the automated capillary ProteinSimple WES system are shown. (C, D) Malondialdehyde (MDA) levels in plasma and GA muscle tissue. (E, F) Advanced oxidation protein product (AOPP) levels in plasma and GA muscle tissue. (G, H) mRNA expression of Nrf2 and catalase in GA muscle. Data are expressed as mean ± SEM. Statistical analysis was performed using one-way ANOVA followed by Tukey's post hoc test. ∗P < 0.05, ∗∗P < 0.01, ∗∗∗P < 0.001 vs. Dex group; #P < 0.05, ##P < 0.01, ###P < 0.001 vs. Control group. Dex, dexamethasone; HMP5/HMP50/HMC50 indicates HMPA (5 or 50 mg/kg BW) or HMCA (50 mg/kg BW); Cbl-b, casitas B-lineage lymphoma proto-oncogene-b; IRS-1, insulin receptor substrate-1; Nrf2, nuclear factor erythroid 2–related factor 2; MDA, malondialdehyde; AOPP, advanced oxidation protein product.

Compared with the vehicle-treated control mice, Dex treatment significantly increased the mRNA and protein expression of Cbl-b (Fig. 4A and B). Cbl-b negatively regulates IRS-1 by degradation through ubiquitination. This reduces the levels of IRS-1 in the cell, thereby impairing the insulin signaling cascade. Therefore, we also measured the level of IRS-1 protein, revealing that Dex treatment significantly decreased IRS-1 compared with the vehicle treatment in the control mice (Fig. 4B). Treatment with the higher dose of HMPA and HMCA effectively suppressed the Dex-induced increase in Cbl-b mRNA and protein expression and upregulated IRS-1 protein expression (Fig. 4A and B). These results suggested that HMPA and HMCA mitigated the oxidative stress-sensitive Cbl-b-mediated protein degradation pathway.

Because Cbl-b is sensitive to oxidative stress and is upregulated under increased levels of reactive oxygen species (ROS), we measured the levels of oxidative stress markers and antioxidants in the plasma and GA muscle samples. MDA is a marker of oxidative stress and a lipid peroxidation product, and AOPPs are markers of oxidative stress [24]. Compared with the vehicle-treated control mice, the MDA level was significantly increased in both the plasma and GA muscle samples of mice treated with Dex (Fig. 4C and D) and was notably attenuated by HMPA and HMCA treatment, particularly in the GA muscle tissue. Similarly, Dex treatment increased the level of AOPPs in both the plasma and GA muscle tissue, which were effectively suppressed by HMPA and HMCA treatment (Fig. 4E and F). Moreover, the mRNA expression of nuclear factor erythroid 2-related factor 2 (Nrf2) was upregulated in response to the Dex treatment (Fig. 4G) compared with the vehicle treatment and was followed by increased levels of catalase, an oxidant-neutralizing enzyme (Fig. 4H). HMPA and HMCA treatment decreased the levels of Nrf2 and catalase expression in the Dex-treated group (Fig. 4G and H). Collectively, these results support HMPA and HMCA as antioxidative compounds capable of suppressing oxidative stress and its effects.

### 3.7 Effect of HMPA and HMCA on FoxO3a and Akt levels

**Fig. 5:**
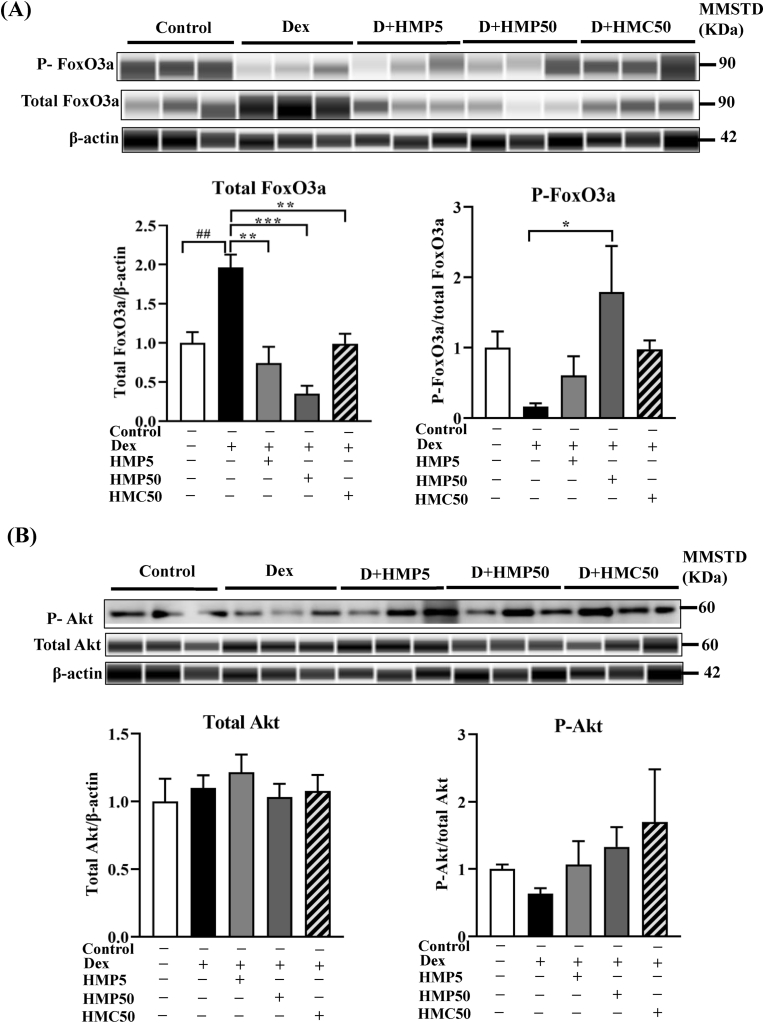
HMPA and HMCA prevented Dex-induced activation of transcription factors and co-factors. Mice were treated as described in Fig. 1, and the protein levels of total and phosphorylated FoxO3a, as well as its upstream effector Akt, were measured by Western blotting. (A) Protein levels of total and phosphorylated FoxO3a. (B) Protein levels of total and phosphorylated Akt. Representative lane views obtained from the automated capillary ProteinSimple WES system are shown. P-Akt was performed by manual western blotting due to the robust and authentic band appearance. Data are expressed as mean ± SEM (n = 3/group). Statistical analysis was performed using one-way ANOVA followed by Tukey's post hoc test. ∗P < 0.05, ∗∗P < 0.01, ∗∗∗P < 0.001 vs. Dex group; #P < 0.05, ##P < 0.01, ###P < 0.001 vs. Control group. Dex, dexamethasone; HMP5/HMP50/HMC50 indicates HMPA (5 or 50 mg/kg BW) or HMCA (50 mg/kg BW); Akt, protein kinase B; FoxO3a, Forkhead box O3a; MMSTD, molecular mass standard.

We investigated the levels of total and phosphorylated FoxO3a, a transcription factor downstream of KLF15 that acts as an upstream regulator of Atrogin-1 and MuRF-1. FoxO3a is also activated during the impairment of IGF-1 signaling caused by Cbl-b ubiquitin ligase [12]. Compared with the control group, total FoxO3a was significantly increased following Dex treatment, whereas the expression of phosphorylated FoxO3a was reduced (Fig. 5A). Treatment with HMPA and HMCA significantly decreased the expression of total FoxO3a. Moreover, the higher dose of HMPA (50 mg/kg BW) effectively enhanced FoxO3a phosphorylation, indicating a reversal of the Dex-mediated activation of FoxO3a. Because phosphorylation negatively regulates FoxO3a, the above results indicated that Dex increased the levels of ubiquitin ligases by dephosphorylating FoxO3a, whereas HMPA counteracted the Dex-mediated effects by enhancing FoxO3a phosphorylation.

FoxO3a activity is tightly controlled by upstream signaling pathways, particularly the Akt/mTOR pathway [25]. Therefore, we measured the protein expression levels of both total and phosphorylated Akt. Although there was no noticeable difference found in total Akt levels, Dex treatment decreased Akt phosphorylation compared with the control group (Fig. 5B). Interestingly, treatment with the higher dose of HMPA and HMCA stimulated Akt phosphorylation almost 2-3 folds compared to Dex, indicating FoxO3a sequestration for nuclear translocation. These findings suggested that HMPA and HMCA regulated FoxO3a modulation to attenuate ubiquitin ligase expression responsible for the induction of muscle atrophy.

## 4 Discussion

We investigated the potential anti-atrophic effects of HMPA and its parent compound HMCA against Dex-induced skeletal muscle atrophy and its possible mechanism of action in mice. The administration of HMPA and HMCA for 21 days significantly attenuated Dex-induced muscle protein degradation and loss in glycolytic muscles, such as the GA and TA. The muscle atrophy-causing ubiquitin ligases Atrogin-1, MuRF-1, and Cbl-b were significantly suppressed by HMPA/HMCA administration. Similarly, the upstream regulators of ubiquitin ligase activation, such as FoxO3a and KLF15, were significantly attenuated by HMPA/HMCA compared with the Dex-treated group.

Glucocorticoids mediate muscle atrophy via the UPS through ubiquitination of the target protein by the ubiquitin ligases Atrogin-1 and MuRF-1 [26]. The activation of the transcription factor KLF15 by Dex activates the UPS, triggering muscle protein breakdown [7]. Moreover, during Dex-induced catabolism, the suppression of Akt/mTOR signaling dephosphorylates FoxO3a [27], facilitating its nuclear translocation. This translocation upregulates muscle-specific E3 ubiquitin ligases, including Atrogin-1 and MuRF-1, contributing to muscle atrophy. In this study, HMPA and HMCA administration ameliorated the Dex-induced upregulation of Atrogin-1, MuRF-1, and KLF15. Similarly, the Dex-mediated induction of FoxO3a was reversed by HMPA and HMCA by stimulating Akt phosphorylation, thereby inhibiting its nuclear translocation and the downstream induction of ubiquitin ligases. Based on these findings, we suggest that both HMPA and HMCA prevent muscle atrophy *in vivo* by inhibiting the expression of ubiquitin ligases through KLF15 and FoxO3a modulation. Furthermore, our *in vivo* results agree with our previously published reports demonstrating the effect of HMPA on C2C12 myotubes [15].

Oxidative stress is caused by an imbalance between pro-oxidants and antioxidants. It plays a major role in muscle atrophy via various mechanisms, including increased ROS production, proteolytic pathway activation, mitochondrial dysfunction, and impaired muscle regeneration [28]. Similarly, elevated ROS levels damage cell proteins, lipids, and DNA, which impair muscle function and contribute to atrophy [29]. Oxidative stress also activates the UPS, which is responsible for protein degradation in muscle cells [30]. It impairs satellite cell function, hindering the recovery of muscles from injury or atrophy [31]. In this study, we measured the levels of MDA and AOPP to assess the extent of oxidative stress. MDA is primarily formed during the peroxidation of polyunsaturated fatty acids (PUFAs) in cell membranes. When PUFAs are oxidized by ROS, this triggers a series of reactions that ultimately lead to MDA production [32,33]. Similarly, AOPPs are formed when proteins undergo oxidative modifications due to ROS activity. AOPPs are considered markers of protein oxidation because they are typically generated during oxidative stress [34]. Both MDA and AOPPs were significantly increased in the plasma and muscle tissue of mice treated with Dex compared with the control group, whereas HMPA/HMCA treatment significantly reduced MDA and AOPP levels (Fig. 4C and D). Moreover, the mRNA expression of Nrf2, which is responsible for attenuating oxidative damage in tissues, was enhanced in parallel with the expression of catalase in Dex-treated mice. These findings suggest an early protective response against oxidative stress. The reduction of Nrf2 and catalase expression by HMPA or HMCA likely reflects a lowered oxidative burden, diminishing the cellular need for compensatory antioxidant gene activation. This interpretation aligns with previous studies showing that effective ROS-lowering interventions can normalize Nrf2 signaling once the oxidative trigger is removed [35,36]. In our study, Dex treatment elevated oxidative damage and upregulated Nrf2 and catalase expression, consistent with an early compensatory antioxidant response. HMPA and HMCA treatment reduced MDA and AOPP levels and concomitantly brought Nrf2 and catalase expression back toward control levels. This suggests that the upstream oxidative stress was alleviated, thereby reducing the demand for Nrf2-mediated transcriptional activation. Future studies quantifying Nrf2 nuclear translocation, ARE-target protein activity, glutathione redox state, and time-course responses will be essential to determine whether this normalization reflects true stress resolution or involves direct modulation of antioxidant signaling pathways.

Consistent with the above results, the mRNA and protein expression of Cbl-b, an oxidative stress-sensitive ubiquitin ligase, was significantly increased following Dex treatment. IGF-1 signaling is a critical regulator of muscle mass and function that protects against muscle atrophy. Cbl-b impairs the IGF-1 signaling pathway by degrading IRS-1, resulting in FoxO3a dephosphorylation and increased levels of Atrogin-1 and MuRF-1. In our study, increased Cbl-b expression degraded IRS-1 (Fig. 4B). Interestingly, HMPA and HMCA effectively reduced the Dex-induced Cbl-b expression and upregulated the level of IRS-1, leading to decreased Atrogin-1 and MuRF-1 expression. Thus, the beneficial effects of HMPA and HMCA also include preventing oxidative stress by suppressing Cbl-b and Cbl-b-mediated IRS degradation.

Glucocorticoids, such as Dex, exert a more pronounced effect on fast-twitch muscle fibers than slow-twitch muscle fibers [37]. These differential effects are attributed to differences in muscle fiber composition, receptor density, metabolic pathways, atrophy susceptibility, and regulatory mechanisms. MuRF-1 also shows specificity in the breakdown of MyHC during Dex-induced atrophy [38]. In our study, Dex treatment significantly decreased fast-type MyHC but did not cause any noticeable changes in slow-type MyHC. Both HMPA and HMCA efficiently attenuated the Dex-induced decrease in fast-type MyHC, possibly due to MuRF-1 suppression by HMPA.

The 1-methyl-L-His and 3-methyl-L-His are two methylated derivatives of the amino acid histidine. 1-(or *N*_τ_)*-*methyl-L-His is primarily associated with muscle tissue, which is released into the bloodstream during muscle catabolism and is considered a potential marker of muscle protein breakdown [39]. Thus, an elevated level of 1-methyl-L-His typically indicates the increased muscle catabolism that occurs during muscle wasting. In contrast, the level of 3-methyl-L-His can be influenced by protein diet and other factors. Food intake like chicken, meat, fish and their products modulate the level of 3-methyl-L-His [40]. 3-methyl-L-His may be increased with 1-methyl-L-His during muscle tissue turnover, as traditionally their detection chromatogram lies in the similar position, making it hard to differentiate between them [41]. However, in our current study, chromatograms are well differentiated from each other (see supplementary figure). Moreover, protein contents in the used chaw food were similar in all the groups, thus, we suggest that due to uniform protein contents in the food, 3-methyl-L-His was unchanged and shows a similar trend (Fig. 2B). However, 1-methyl-L-His was significantly changed, which could be considered a real marker for protein catabolism induced by Dex. Consistently, in our study, Dex treatment significantly increased the 1-methyl-L-His level compared to the control group. Interestingly, HMPA and HMCA treatment effectively attenuated the level of 1-methyl-L-His. These results further suggest that HMPA/HMCA are effective against Dex-induced muscle protein catabolism.

HMPA and HMCA are related phenolic compounds with distinct chemical structures and biological activities. HMPA is a reduced form of HMCA with an additional two hydrogen atoms typically involving the saturation of double bonds present in HMCA. HMCA is more extensively studied and widely used in various industries due to its well-documented health benefits [42], while HMPA is still undergoing extensive research for its potential health benefits [15,16,18,19]. In our study, both HMPA and HMCA were found to be effective against muscle atrophy; however, whether the effect of HMCA is attributable to its metabolite, HMPA, requires further extensive research. Interestingly, Kitano et al. reported that the antiobesity effect of HMCA was due to its metabolic entity HMPA [17]. Similarly, they found that interruption of conversion of HMCA into HMPA using antibiotics abolished the hypolipidemic effects of HMCA.

Detailed pharmacokinetic and toxicological studies of HMPA are scarce. Abe et al. [43] reported the pharmacokinetic properties of HMPA using Sprague–Dawley rats. Following the administration of HMPA (10 mg/kg BW), they detected intact and conjugated HMPA in the bloodstream, peaking in concentration after 15 min, and in the target organs 6 h after administration, indicating their rapid conversion into respective conjugates. Another study reported that the maximum plasma concentration of HMPA was attained 5–6 h after polyphenol intake [44]. HMPA bioavailability is often limited due to its rapid metabolism and conjugation. Therefore, HMPA is considered to show moderate bioavailability, which is influenced by factors such as absorption, metabolism, and excretion. Moreover, HMPA has a low toxicity profile, particularly when consumed in dietary amounts. However, in-depth studies are necessary to fully elucidate its safety profile and potential toxicological effects, particularly at higher doses or in specific populations.

Despite the promising findings, this study has some limitations. First, HMCA was administered only at a high dose (50 mg/kg), which limits the ability to assess its dose-dependent effects in parallel with HMPA. Future studies should explore lower doses of HMCA to better define its pharmacodynamics. Second, although the protective effects of HMPA and HMCA were evident *in vivo*, the relative contribution of direct skeletal muscle action versus indirect systemic mechanisms (e.g., anti-inflammatory or gut-mediated effects) remains unclear. Third, we used only female mice to reduce hormonal confounding, but this precludes insights into potential sex-based differences in glucocorticoid responsiveness. Lastly, while HMPA and HMCA normalized Nrf2 and catalase expression likely due to reduced oxidative stress, the distinction between true stress resolution and direct modulation of antioxidant signaling warrants further investigation.

## 5 Conclusion

We demonstrated that both HMPA and HMCA are capable of preventing Dex-induced muscle atrophy in C57BL/6J female mice. Both compounds downregulated the expression of genes involved in muscle atrophy and reduced the Dex-induced proteolysis of MyHC. Oxidative stress and Cbl-b expression were effectively attenuated by HMPA and HMCA in atrophied muscles. Therefore, we suggest both HMPA and HMCA as novel natural supplements with beneficial effects on muscle atrophy. However, further detailed studies are necessary to determine whether the effect of HMCA is inherent or ascribable to HMPA.

## Authors’ contribution

Anayt Ulla: Conceptualization, Methodology, Investigation, Data curation, Formal analysis, Writing-original draft. Honomi Ogura: Methodology and Formal analysis. MD Mizanur Rahman: Investigation, Data curation, Formal analysis. Saya Nakamura: Investigation and Formal analysis. Yuka Ichiba: Investigation and formal analysis. Haruka Tsuda: Methodology, Data Curation, Formal analysis. Takayuki Uchida: Investigation, Formal analysis. Hiroyuki Kayaki: writing original draft, Formal analysis. Yosuke Nishitani: Methodology, Visualization, Formal analysis. Susumu Yoshino: Conceptualization, Methodology, Visualization, Writing-review & editing. Hiroshige Kuwahara: Methodology, Visualization, Writing-review & editing. Toshiro Matsui: Investigation, Writing-review and editing, Formal analysis. Takeshi Nikawa: Conceptualization, Methodology, Visualization, Project administration, Funding Acquisition, Writing-review and editing, Supervision.

## Funding

This work was supported by Maruzen Pharmaceuticals Co.

## CRediT authorship contribution statement

**Anayt Ulla:** Conceptualization, Data curation, Formal analysis, Investigation, Methodology, Writing – original draft. **Honomi Ogura:** Formal analysis, Methodology. **Md Mizanur Rahman:** Data curation, Formal analysis, Investigation. **Saya Nakamura:** Formal analysis, Investigation. **Yuka Ichiba:** Formal analysis, Investigation. **Haruka Tsuda:** Data curation, Formal analysis, Methodology. **Takayuki Uchida:** Formal analysis, Investigation. **Hiroyuki Kayaki:** Formal analysis, Writing – original draft. **Yosuke Nishitani:** Formal analysis, Methodology, Visualization. **Susumu Yoshino:** Conceptualization, Methodology, Visualization, Writing – review & editing. **Hiroshige Kuwahara:** Methodology, Visualization, Writing – review & editing. **Toshiro Matsui:** Formal analysis, Investigation, Writing – review & editing. **Takeshi Nikawa:** Conceptualization, Funding acquisition, Methodology, Project administration, Supervision, Visualization, Writing – review & editing.

## Declaration of competing interest

The authors declare the following financial interests/personal relationships which may be considered as potential competing interests:Hiroyuki Kayaki, Yosuke Nishitani, Susumu Yoshino and Hiroshige Kuwahara are employed by Maruzen Pharmaceutical Co., Ltd. All other authors have nothing to declare the conflict of interest. This research was supported by Maruzen Pharmaceutical Co., Ltd.

## Data Availability

Data can be shared at reasonable requests to the corresponding author.
