# Supplementary figures and images for: Protective effects of 3-(4-hydroxy-3-methoxyphenyl) propionic acid against dexamethasone-induced muscle atrophy: modulation of associated genes and oxidative stress in female mice
Source: Biochem Biophys Rep. 2026 Feb 9;45:102483. doi: 10.1016/j.bbrep.2026.102483 (PMC12914295; doi:10.1016/j.bbrep.2026.102483)

## Supplementary Figure

### Methyl-L-His Chromatogram

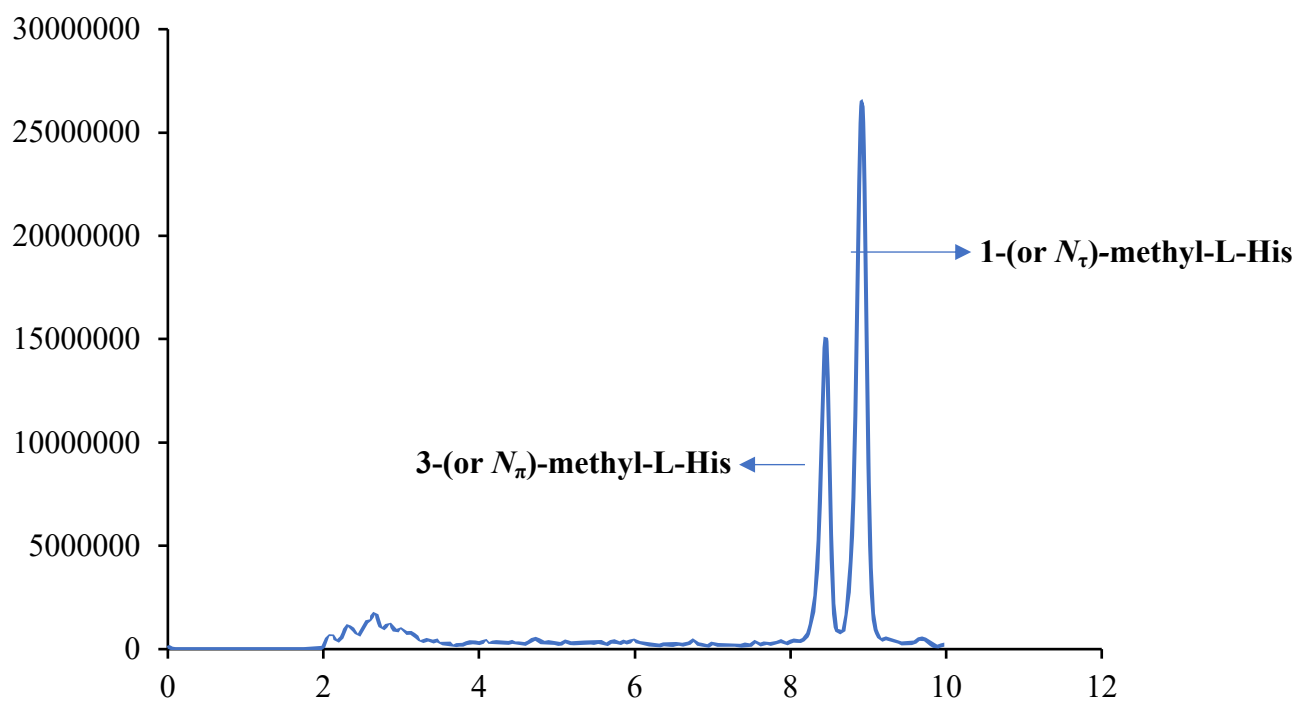

Supplement: Multimedia component 1 [file mmc1.pdf]
