# Supplementary material for: Protective effects of 3-(4-hydroxy-3-methoxyphenyl) propionic acid against dexamethasone-induced muscle atrophy: modulation of associated genes and oxidative stress in female mice
Source: Biochem Biophys Rep. 2026 Feb 9;45:102483. doi: 10.1016/j.bbrep.2026.102483 (PMC12914295; doi:10.1016/j.bbrep.2026.102483)

**Manuscript title:** Protective effects of 3-(4-hydroxy-3-methoxyphenyl) propionic acid against dexamethasone-induced muscle atrophy: modulation of associated genes and oxidative stress in female mice

**Manuscript Number:** BBREP-D-26-00159

**Correspondence:** Dr. Takeshi Nikawa, (nikawa@tokushima-u.ac.jp)

**Technical note:** All proteins were analyzed using the ProteinSimple WES system, except phosphorylated Akt, which was analyzed by conventional SDS–PAGE western blotting, as described in the Methods section.

**Fig. 2(B) of the manuscript Figure**

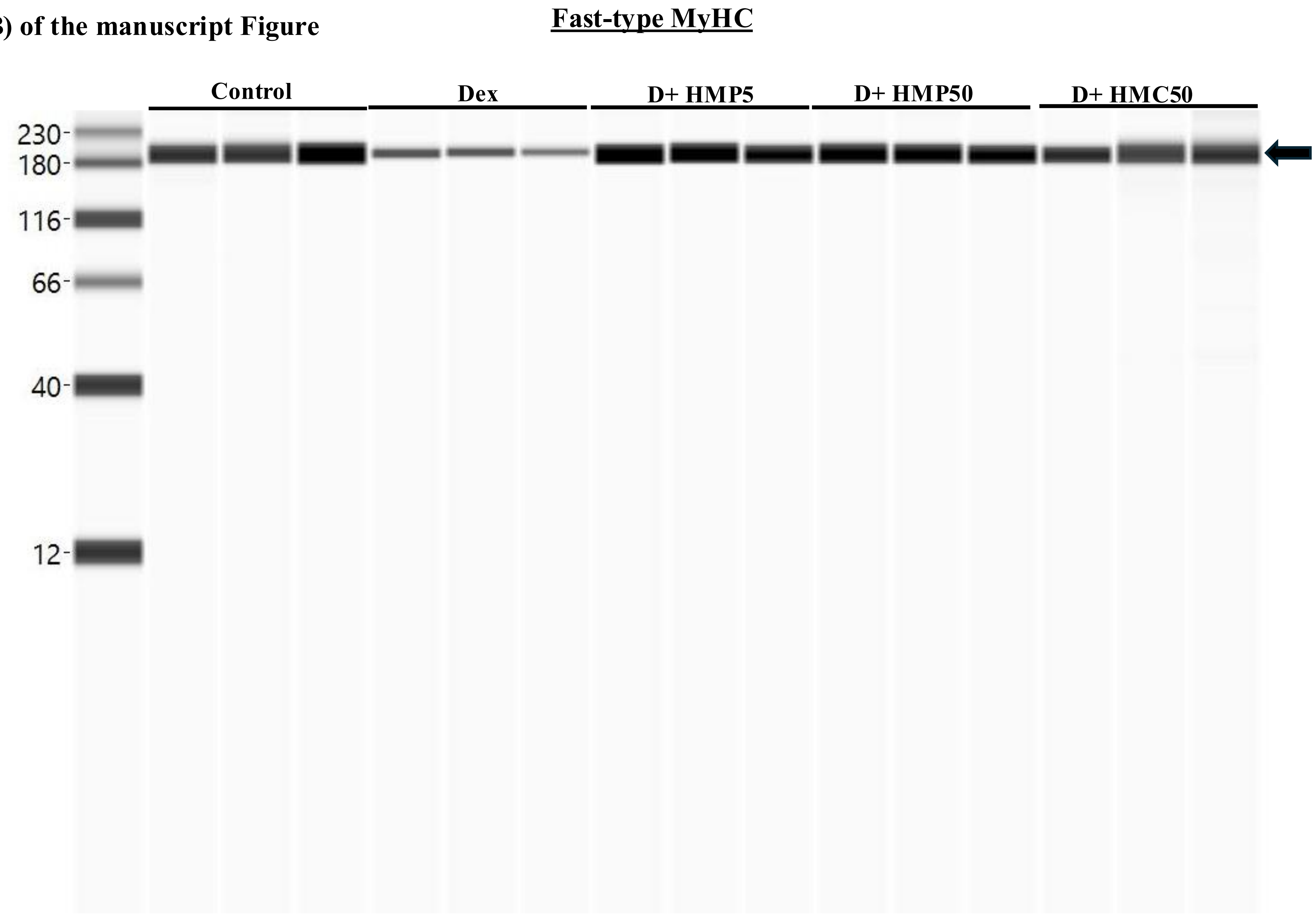

Fig. 2(B) of the manuscript Figure

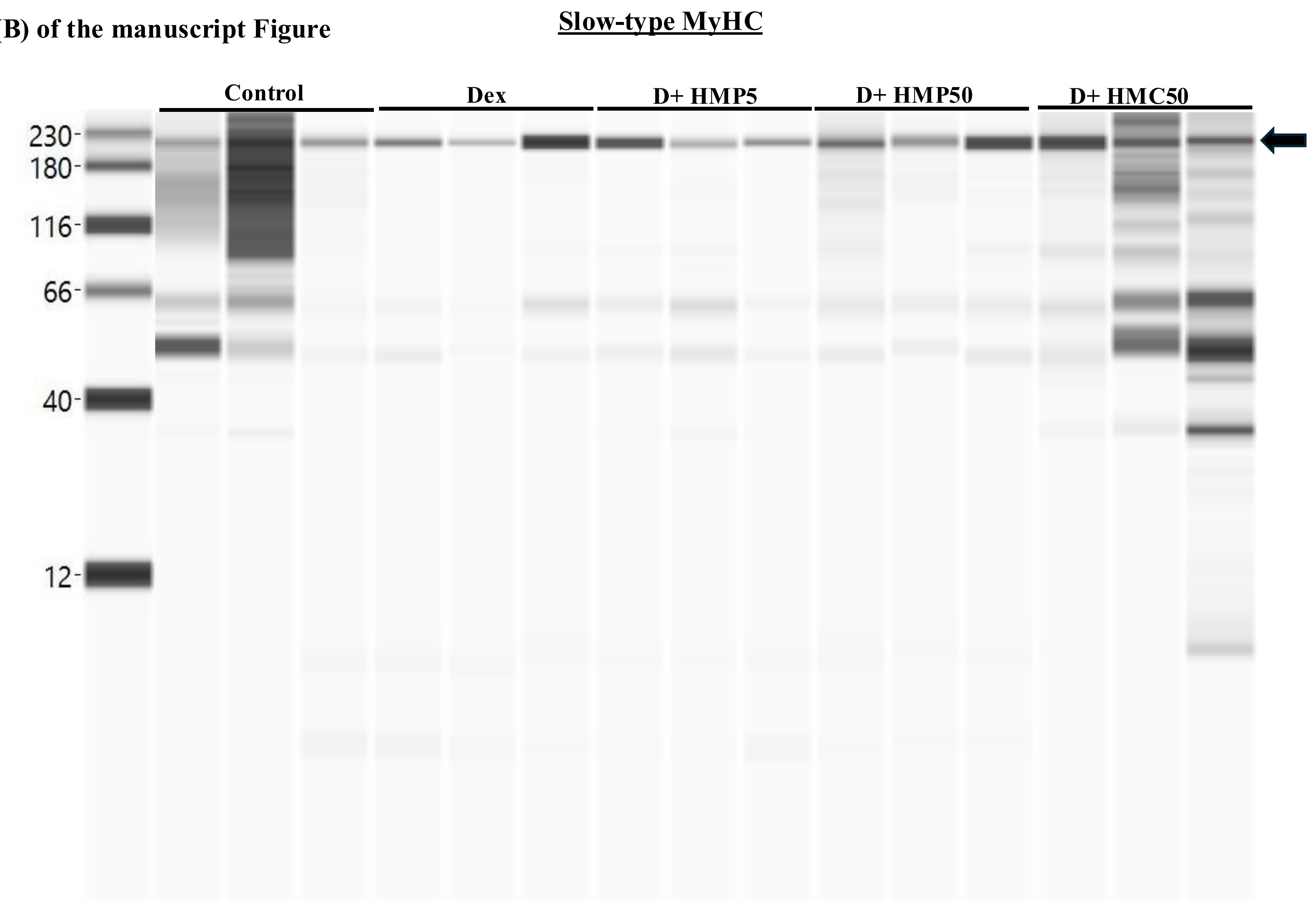

**Fig. 2(B) of the manuscript Figure**

**β-actin for Fast & Slow-type MyHC**

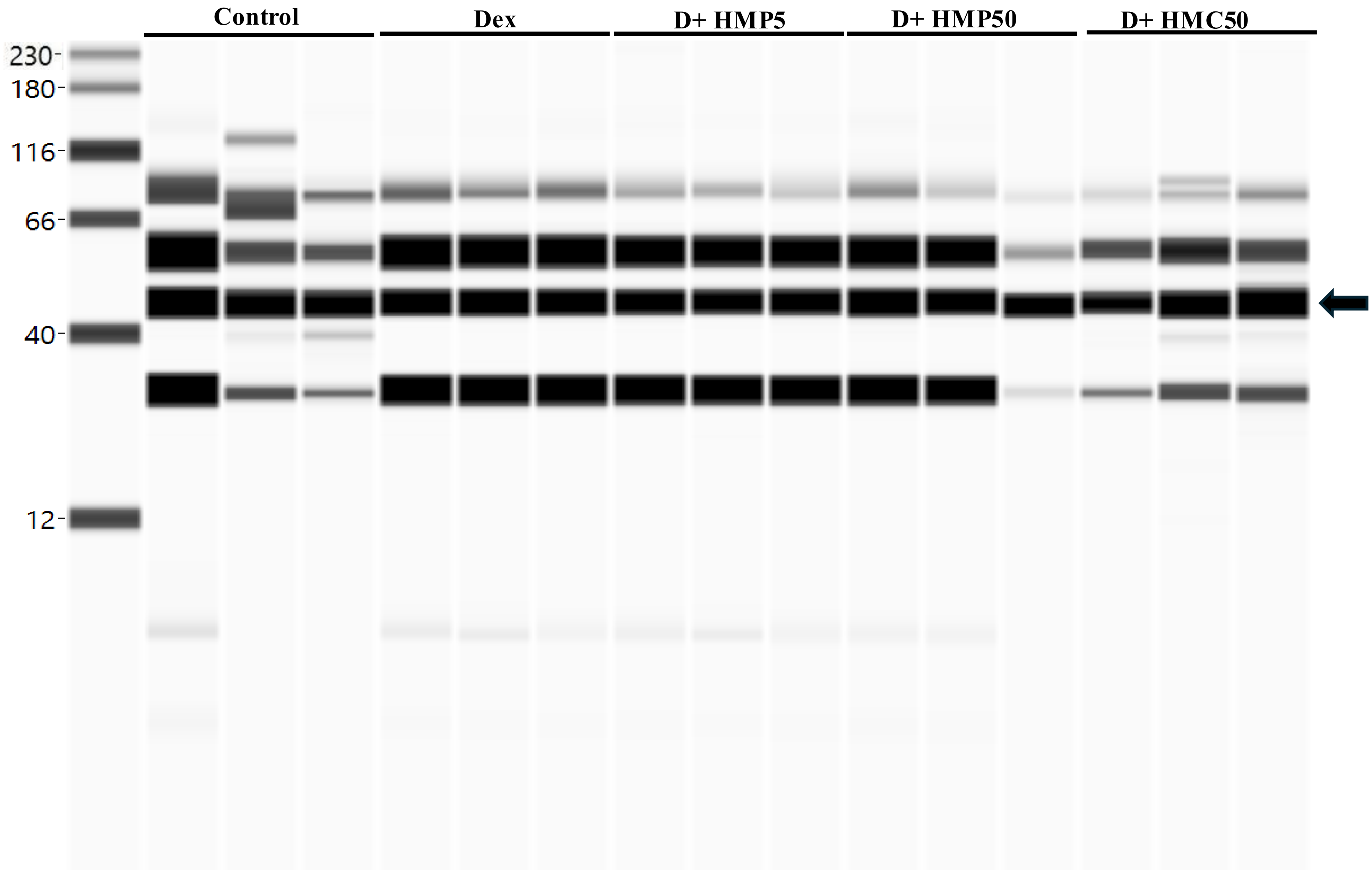

**Fig. 3(D) of the manuscript Figure**

**Atrogin-1**

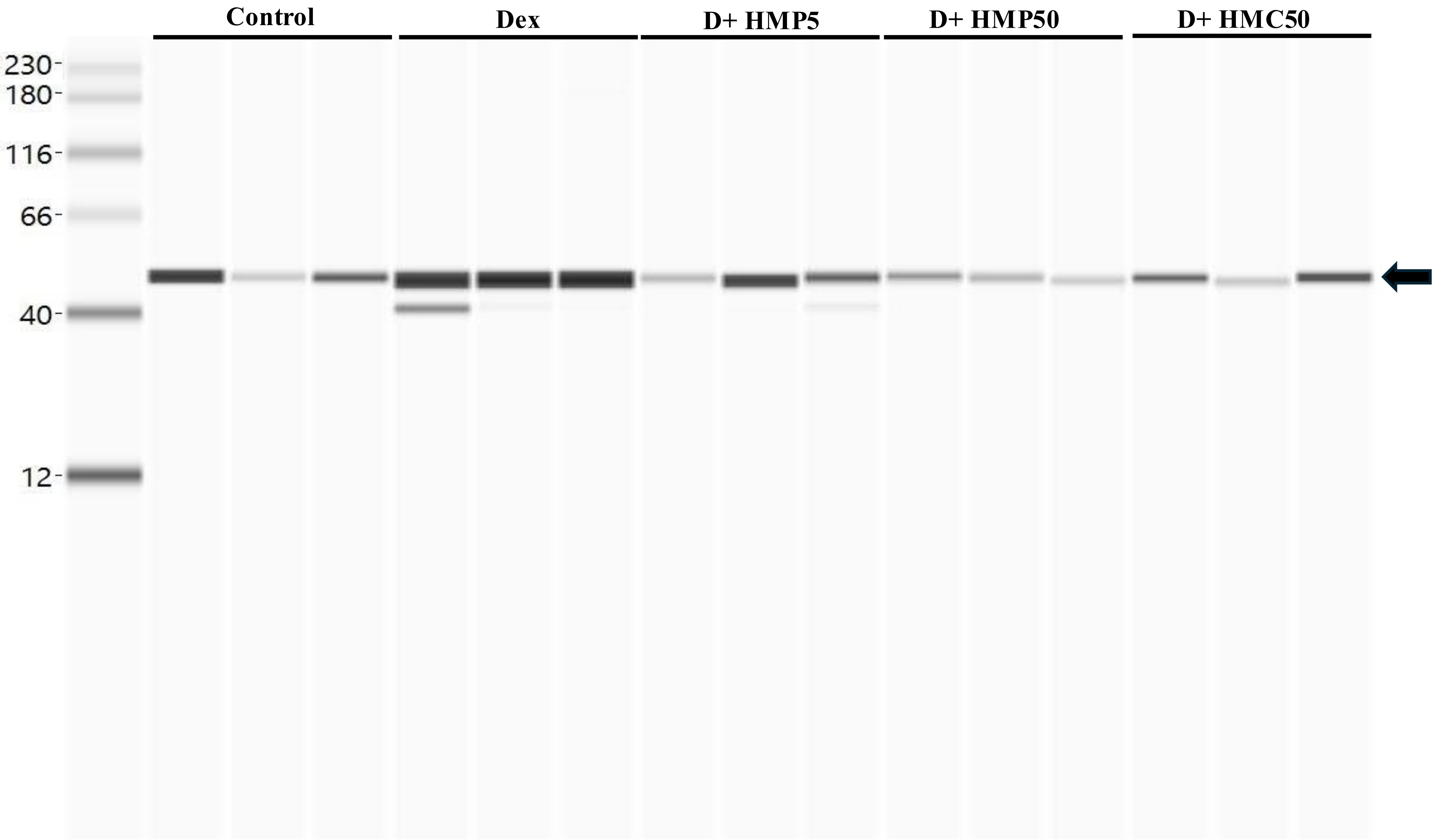

**Fig. 3(D) of the manuscript Figure**

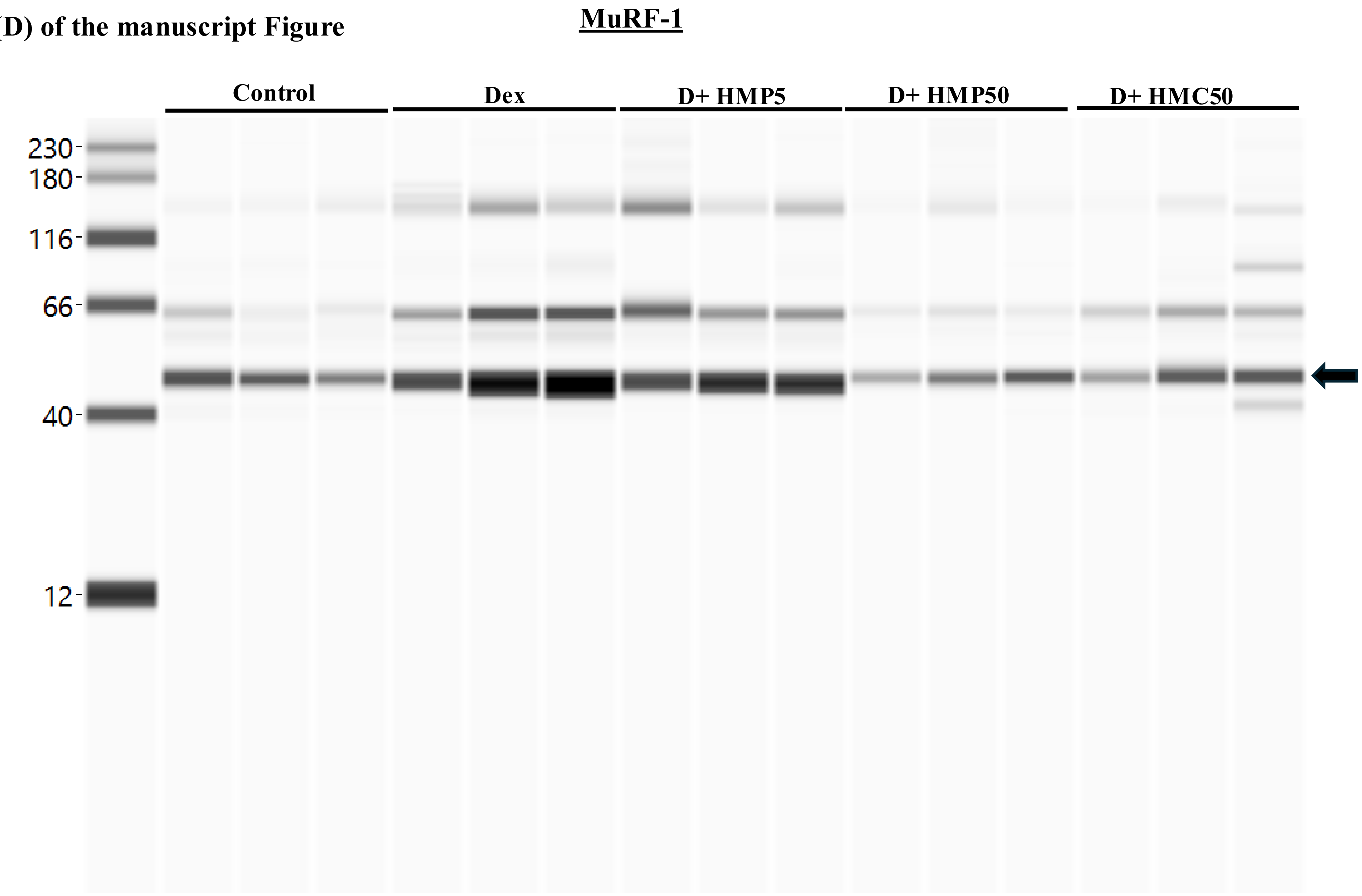

**Fig. 3(D) of the manuscript Figure**

## β-actin for Atrogin-1 & MuRF-1

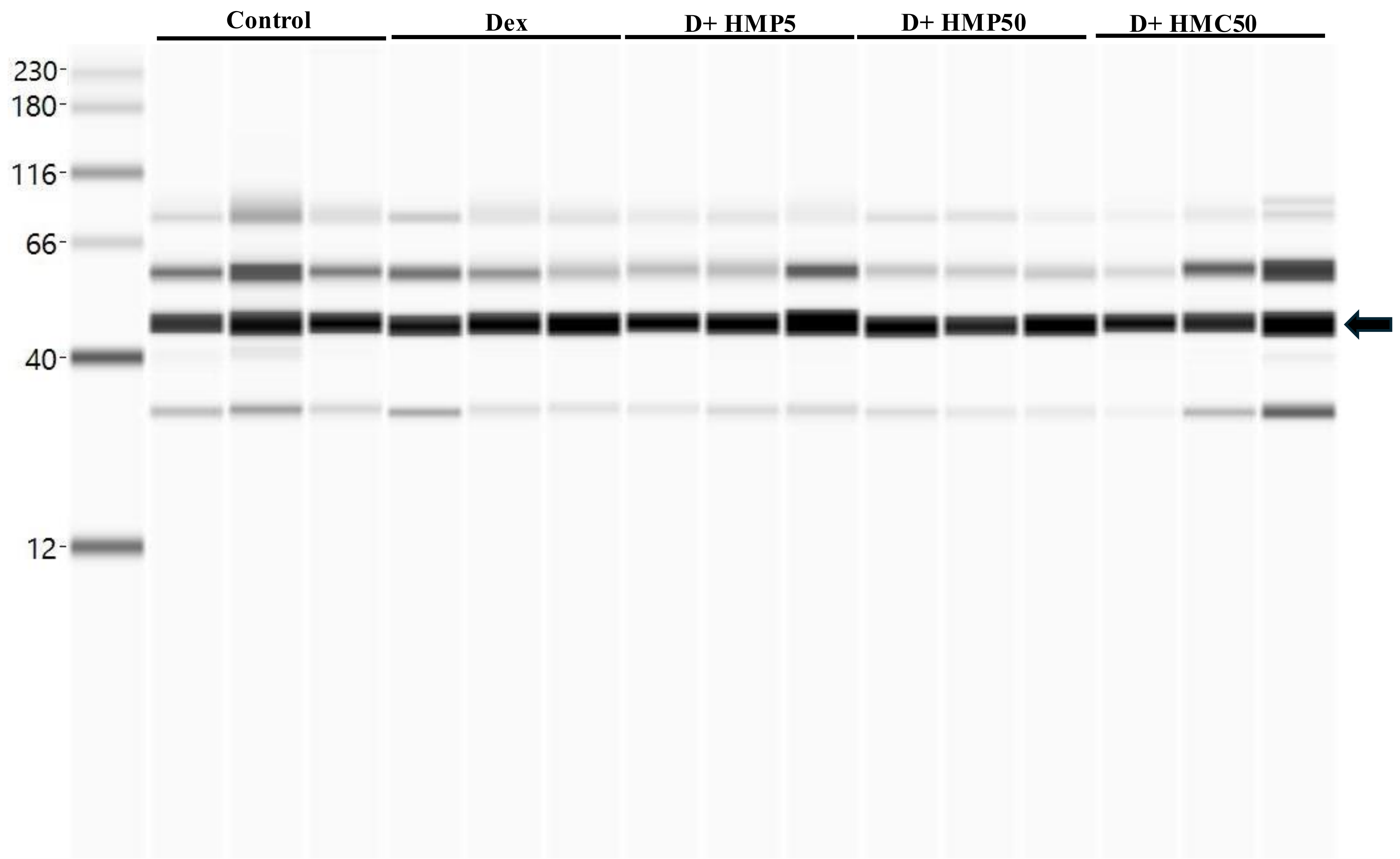

**Fig. 4(B) of the manuscript Figure**

**<u>Cbl-b</u>**

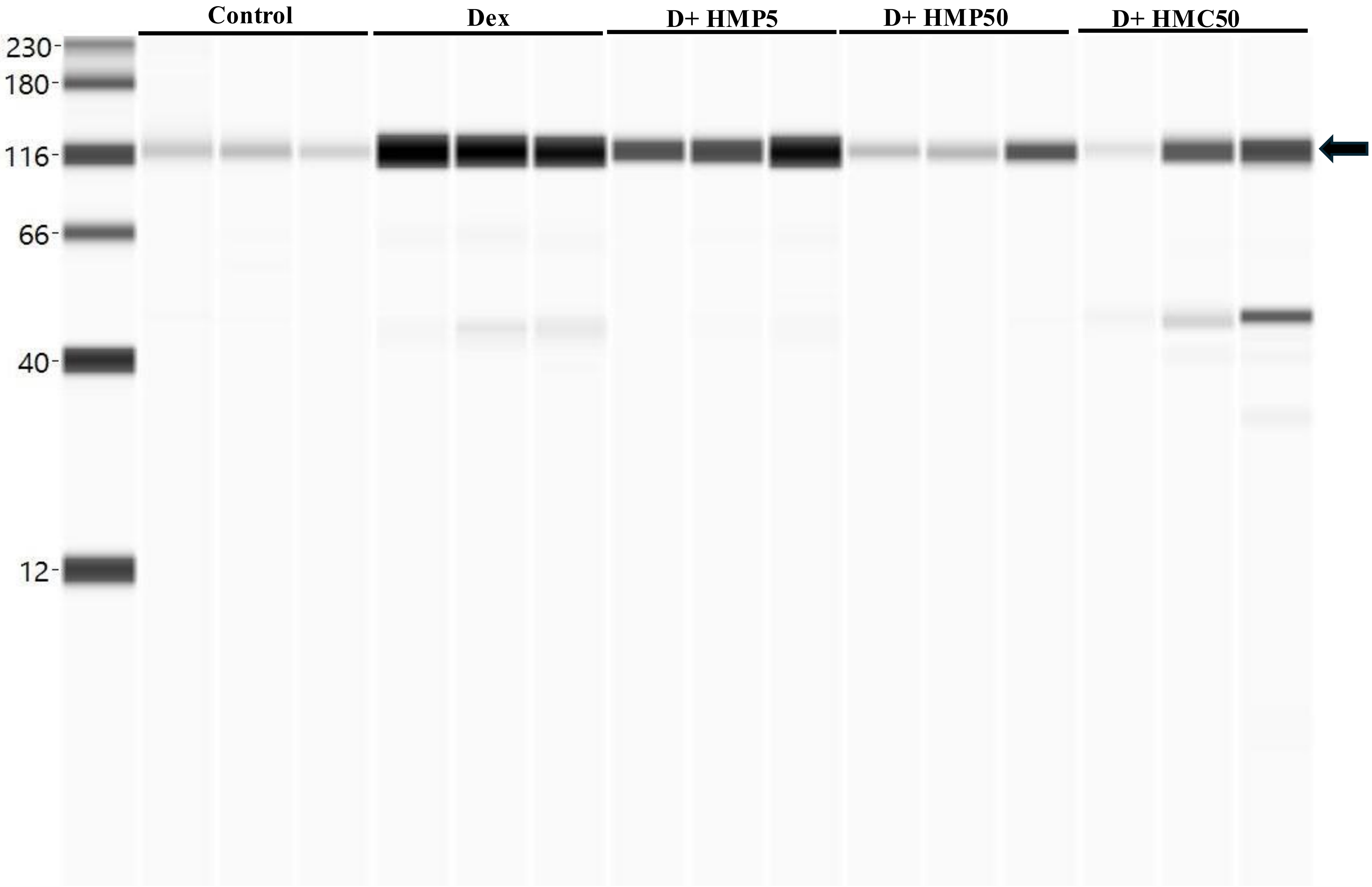

**Fig. 4(B) of the manuscript Figure**

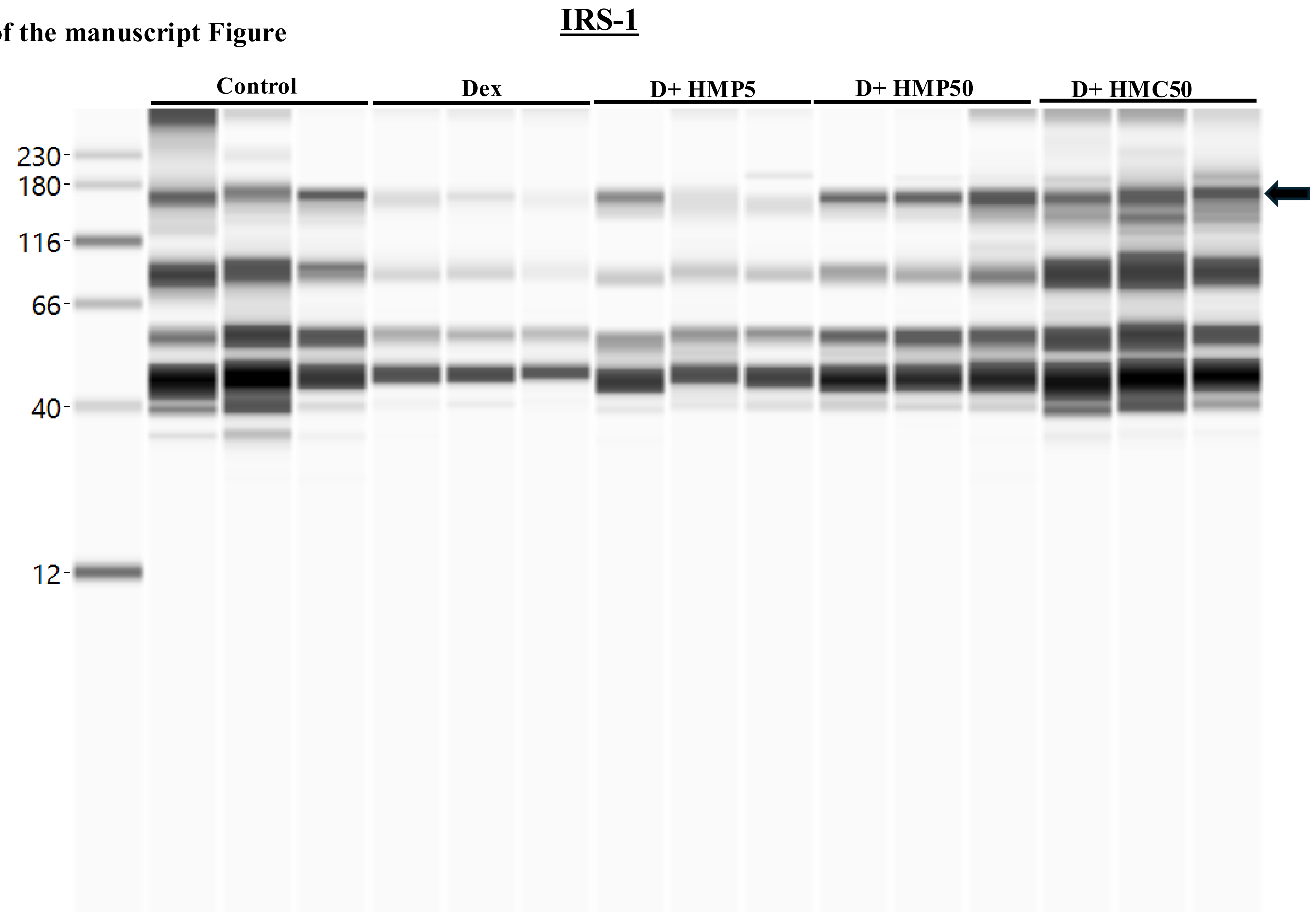

**Fig. 4(B) of the manuscript Figure**

## β-actin for Cbl-b & IRS-1

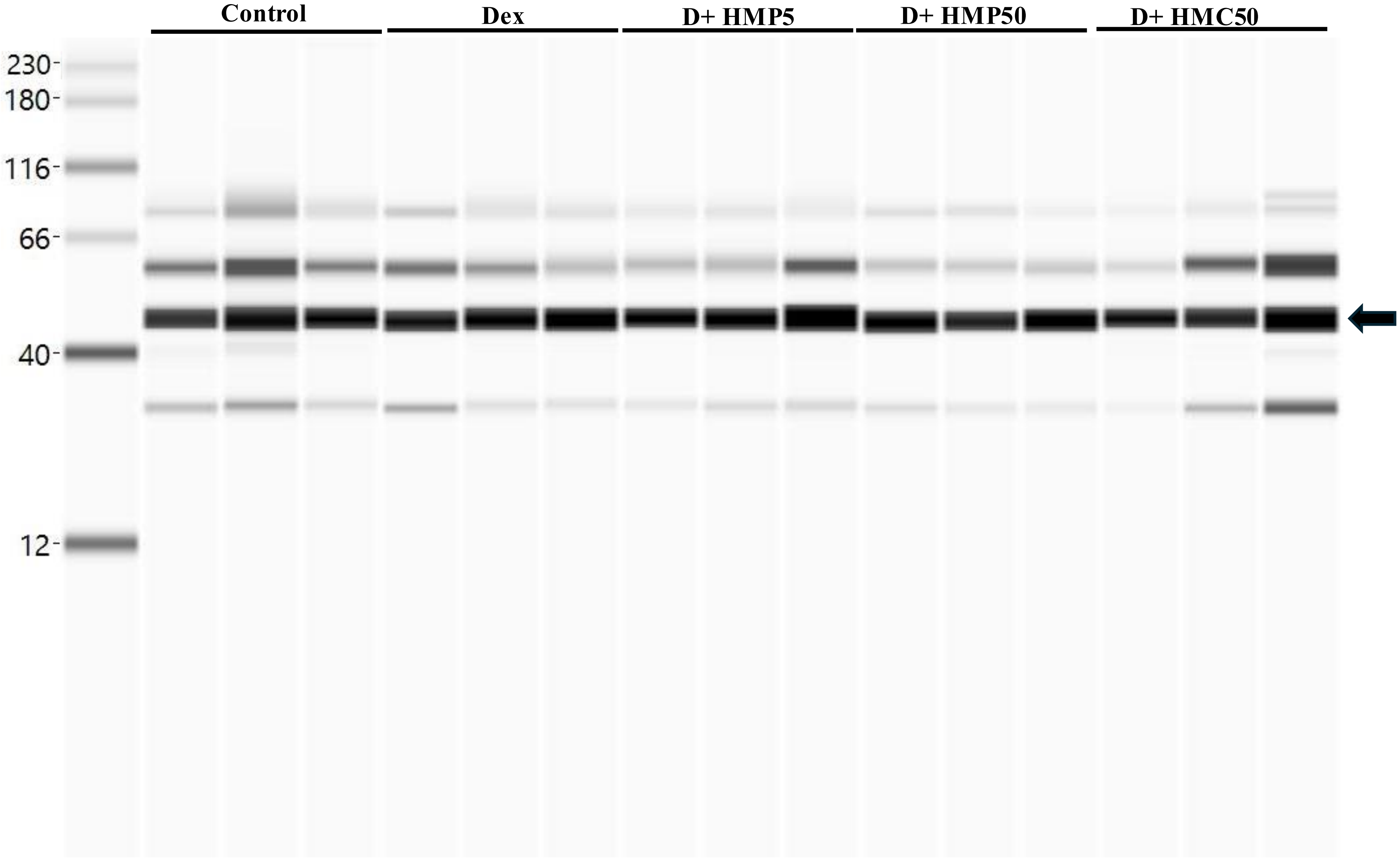

Fig. 5(A) of the manuscript Figure

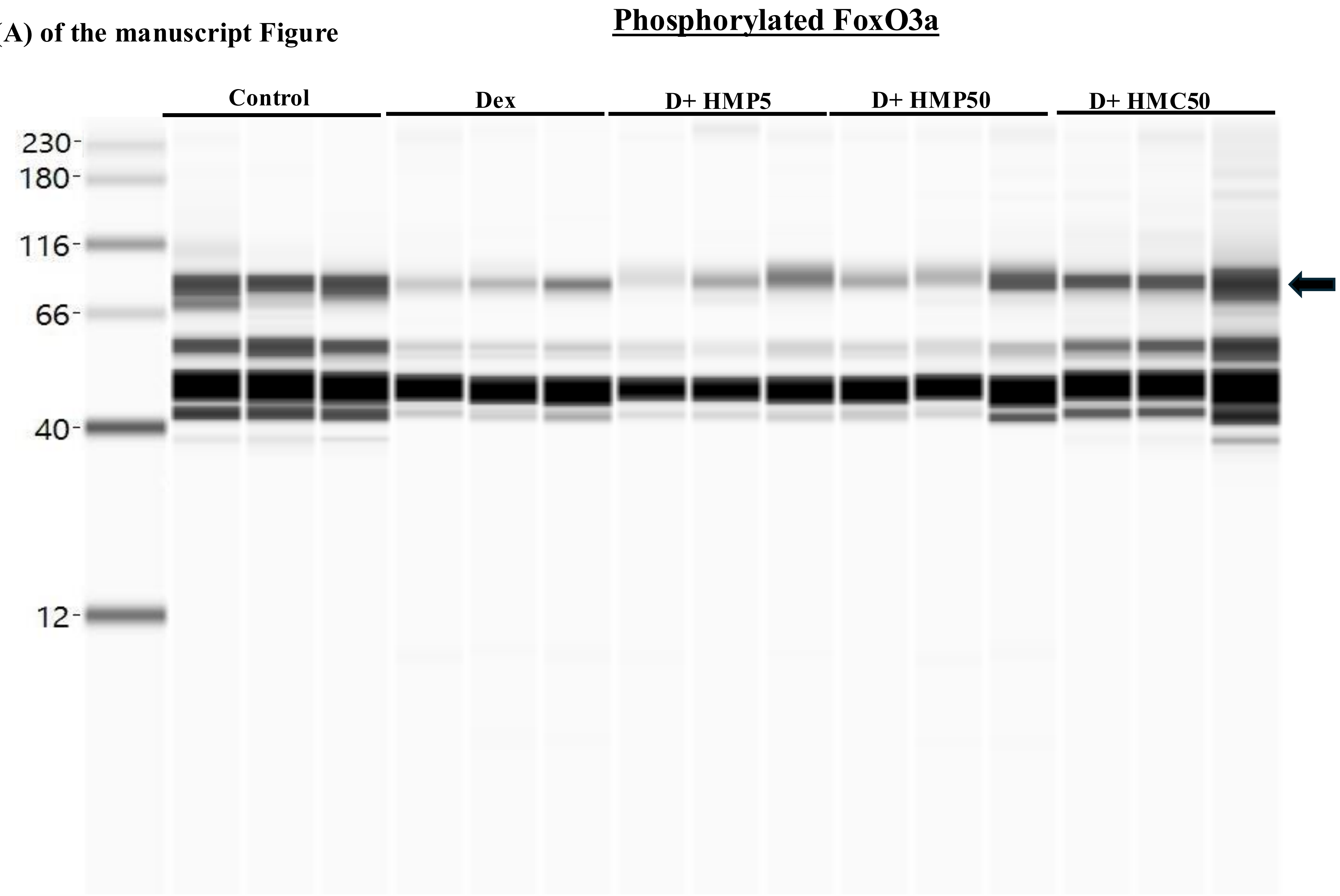

Fig. 5(A) of the manuscript Figure

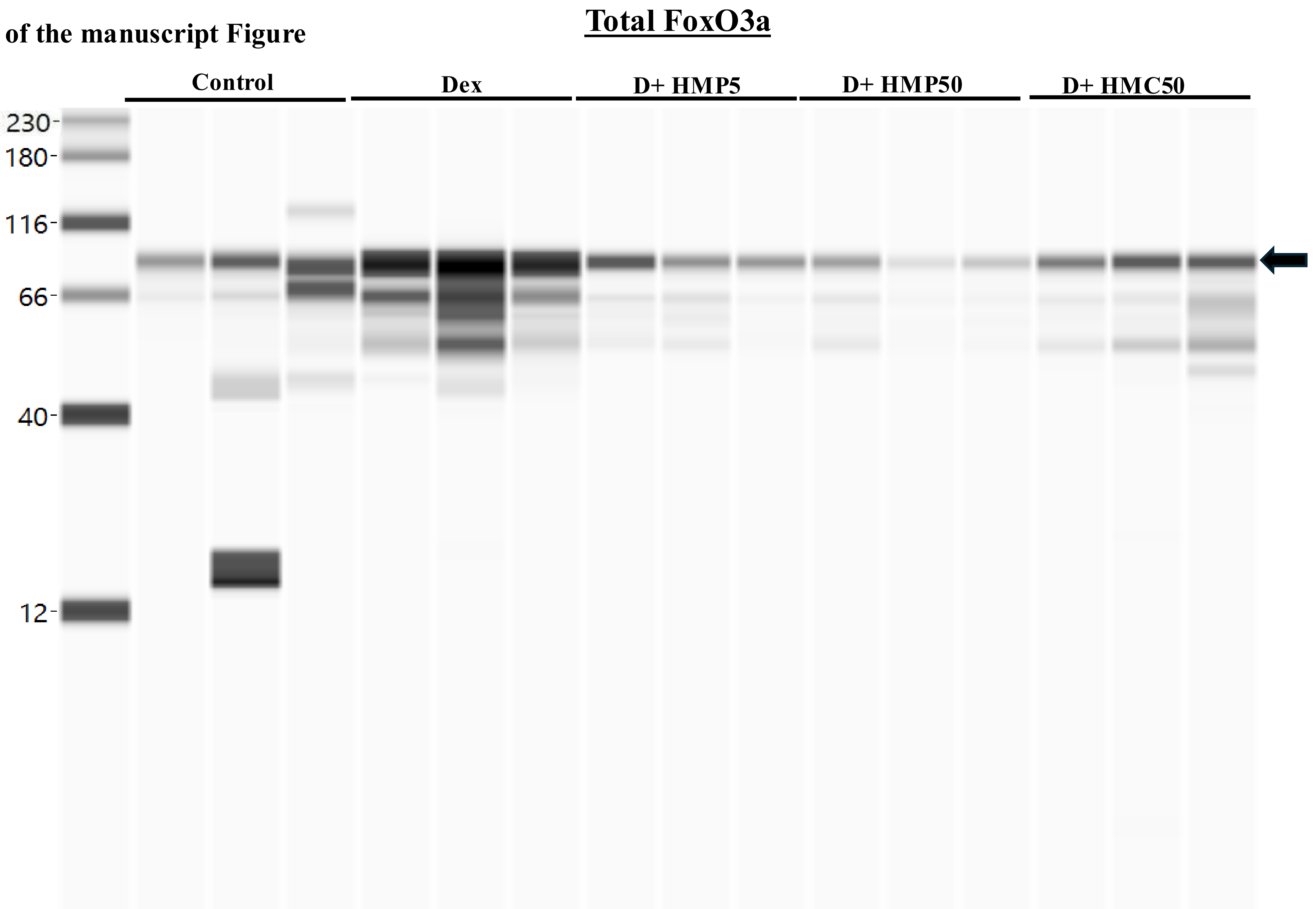

Fig. 5(B) of the manuscript Figure

<u>**Phosphorylated Akt**</u>

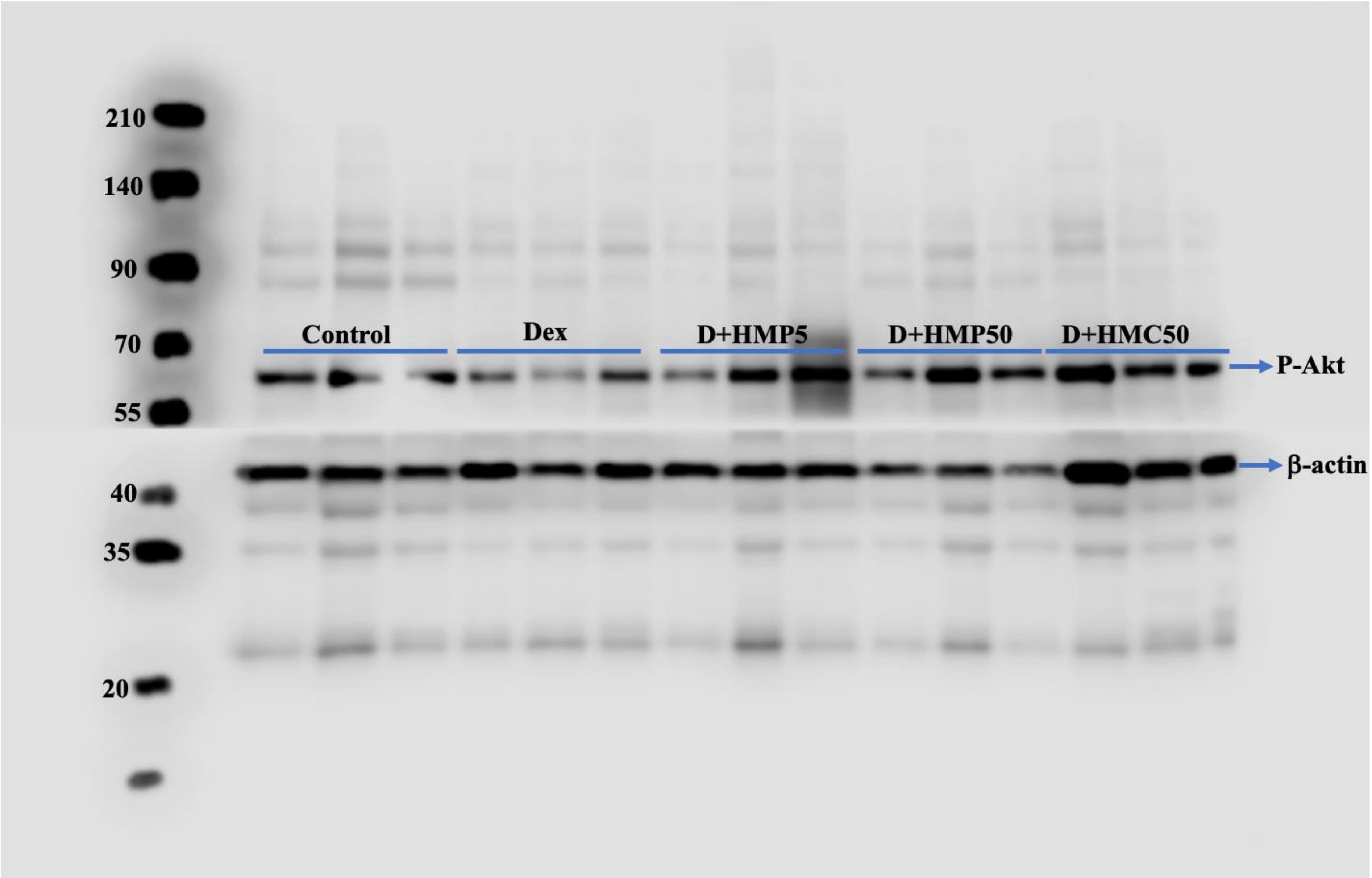

Fig. 5(B) of the manuscript Figure

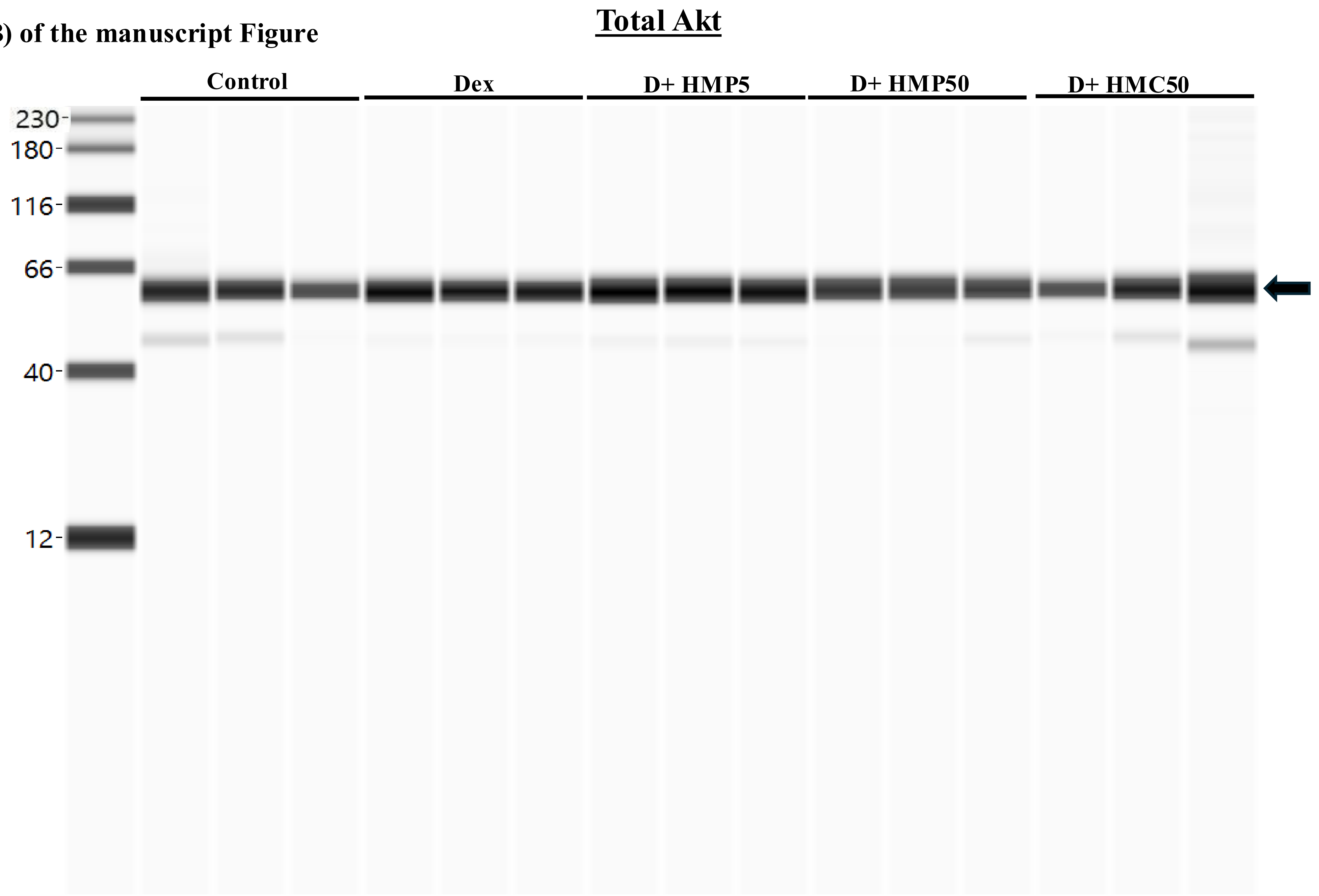

## β-actin for FoxO3a and Akt

**Fig. 5(B) of the manuscript Figure**

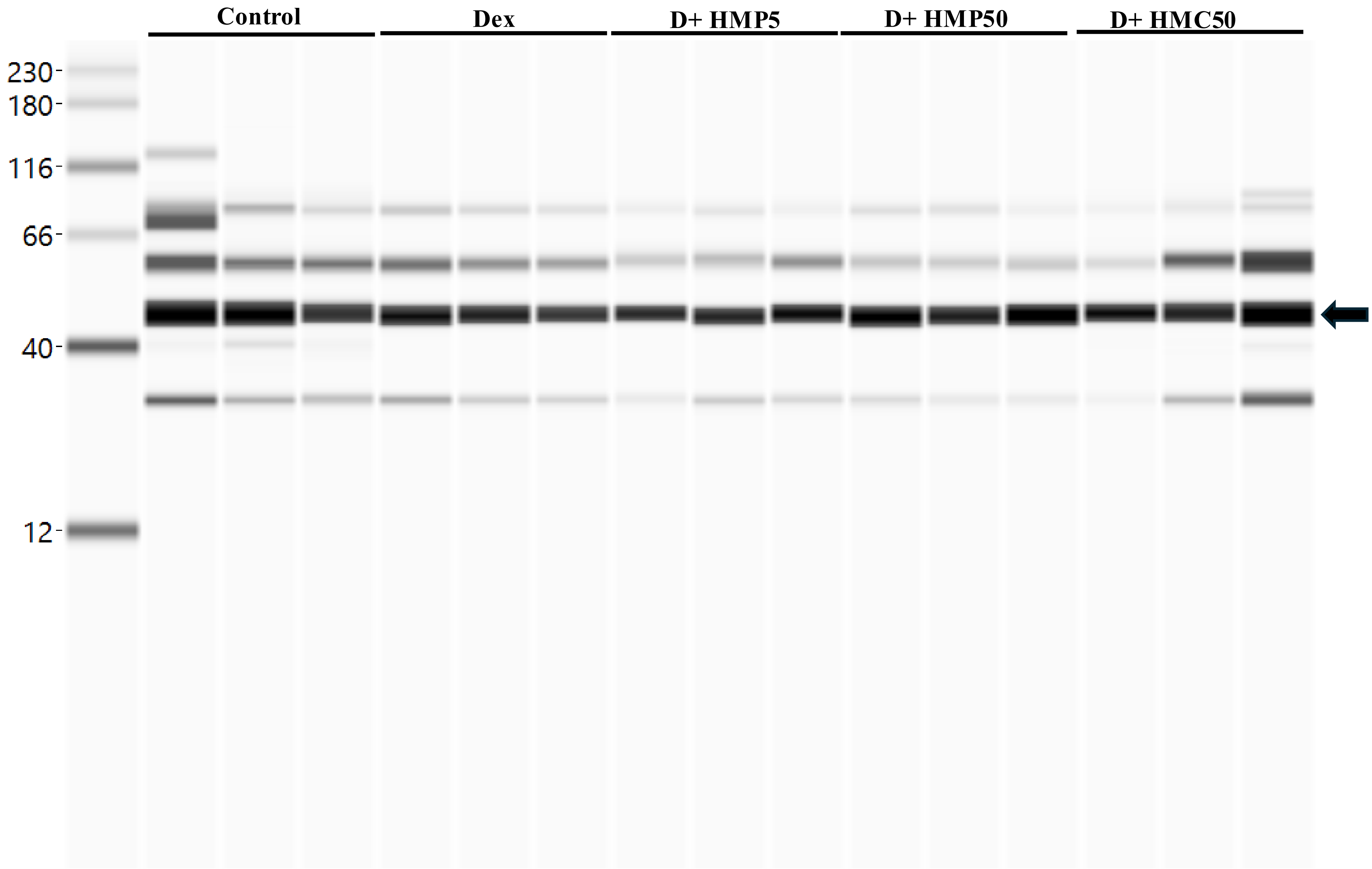

Supplement: Multimedia component 2 [file mmc2.pdf]
